# Borylated 5-Membered Ring Iminosugars: Synthesis and Biological Evaluation for Glycosidase Inhibition and Anticancer Properties for Application in Boron Neutron Capture Therapy (BNCT)—Part 2

**DOI:** 10.3390/ph18111739

**Published:** 2025-11-17

**Authors:** Kate Prichard, Kosuke Yoshimura, Suzuka Yamamoto, Atsumi Taguchi, Barbara Bartholomew, Jayne Gilbert, Jennette Sakoff, Robert Nash, Atsushi Kato, Michela Simone

**Affiliations:** 1 Discipline of Chemistry, University of Newcastle, Callaghan, NSW 2308, Australia; 2 Department of Hospital Pharmacy, University of Toyama, 2630 Sugitani, Toyama 930-0194, Japan; d2362308@ems.u-toyama.ac.jp (K.Y.); m24b1332@ems.u-toyama.ac.jp (S.Y.); atsut@med.u-toyama.ac.jp (A.T.); kato@med.u-toyama.ac.jp (A.K.); 3 PhytoQuest Ltd., Plas Gogerddan, Aberystwyth, Ceredigion SY23 3EB, UK; barbara.bartholomew@phytoquest.co.uk (B.B.); robert.nash@phytoquest.co.uk (R.N.); 4 Department of Medical Oncology, Calvary Mater Newcastle Hospital, Waratah, NSW 2298, Australia; jayne.gilbert@newcastle.edu.au (J.G.); jennette.sakoff@newcastle.edu.au (J.S.)

**Keywords:** monosaccharide, iminosugar, boronic acid, boronate, boron, BNCT, glycosidase, cancer, Fsp^3^ index, NMR, mutarotation, borarotation, induced fit

## Abstract

**Background:** The synthesis and biological investigation of pyrrolidine (L-*gulo*) iminosugars bearing an organic boron pharmacophore in *ortho* and *meta* positions of an *N*-benzyl group is reported. This paper completes the structure–activity relationship data for this novel family of boron-bearing iminosugars. These can establish reversible intramolecular interactions via dative bonding from nucleophilic amino acid side chains to the empty *p*-orbital of the boron atom. **Methods:** Inhibitory activities against two panels of glycosidases and cancer cell lines were investigated to ascertain structure–activity relationship profiles for these novel iminosugar drug leads. **Results:** These iminosugars display selective, moderate-to-weak inhibitions (IC_50_s = 116–617 μM) of β-D-galactosidase (bovine liver), and indications of inhibition of β-D-glucosidases (almond, bovine liver) (IC_50_s = 633 and 710 μM) and α-D-glucosidases (rice, yeast, rat intestinal maltase) (IC_50_s = 106–784 μM). The boronic acid group emerges as a useful pharmacophore for management of lysosomal storage disorders via the chaperone-mediated therapy approach. The cancer assays revealed that the A2780 ovarian carcinoma cell line is selectively inhibited by all compounds screened and the MIA-Pa-Ca2 pancreatic carcinoma cell line is selectively inhibited by most compounds. Growth inhibition and GI_50_ values were most potent for the **meta 7** side-product. **Conclusions:** Beyond the cancer cell line inhibition and dose-response capabilities, the real therapeutic potential of these borylated drugs lies in their switch on/switch off activation under boron neutron capture therapy (BNCT) radiotherapeutic conditions, thus providing an important area of application for borylated monosaccharides.

## 1. Introduction

From a historical perspective, groundbreaking applications have been achieved in boron chemistry in several areas, spanning boranes and organoboranes—leading to the development of the first general asymmetric synthesis of pure enantiomers -, cross-coupling reactions and semiconductors [1,2,3,4]. Currently, boron-10 is a promising isotope in boron neutron capture therapy (BNCT); metal borides possess exceptional physical properties, such as hardness, high melting and boiling points, light-weightness, chemical inertness, and superconductivity, which make them amenable to high-performance environments, including space applications, nuclear reactor safety operations, as rocket/aircraft propellant, component of tank armours, and next-generation electrolytic and fuel-generation systems, to name a few [5,6,7,8,9].

Boron’s chemistry is wonderfully rich, displaying complex chemical equilibria [10,11,12,13,14,15], which make it amenable to varied applications [16,17,18,19,20]. In medicinal chemistry, installation of organic boron pharmacophores on high Fsp^3^ index substrates (e.g., monosaccharides, which contain heteroatoms, many consecutive stereocentres, and anomeric forms) expands the interactions between borylated drugs and biological systems to reversible covalent bonds, changes in shape and electronics at the boron and neighbouring sites. Organic B atoms thus introduce significant structural and electronic refinements to the induced fit model [21,22,23] by providing drug molecules that are imbued with conformational and electronic flexibility to adapt into enzyme active sites and neighbouring sites. Drugs and enzymes team up to work in unison and adapt to each other.

A high Fsp^3^ index [24,25] has been identified as one of the main factors propelling drug leads through to clinical success. We believe that the expansion of the palette of interactions between drug and enzyme is another important factor [26,27,28,29,30]. This paper completes the discussion on the synthesis and biological evaluation of a novel family of borylated iminosugars [30].

Our group has worked in the areas of carbohydrate chemistry [27] and iminosugar [31] research for many years. Here, we communicate the synthesis to borylated 5-membered ring iminosugars of l-*gulo* absolute stereochemical configuration via development of synthetic and purification protocols to several selective, up to moderately potent glycosidase inhibitors and perspective BNCT agents.

This type of drug leads and previous ones we have communicated [27,28,29,30,32,33] are quite unique in the medicinal chemical literature. They introduce a proof-of-concept and open up a new area in medicinal chemistry, namely borylated monosaccharides and derivatives, imbued with low toxicities (thanks to the organic boron moiety), the capacity to mimic carbohydrates and exploit the same entry mechanisms across membranes [34], glycosidase modulation and switch on/switch off BNCT capabilities that allows cancer management via a milder radiation therapy methodology.

## 2. Results and Discussion

### 2.1. Synthesis

**Scheme 1:**
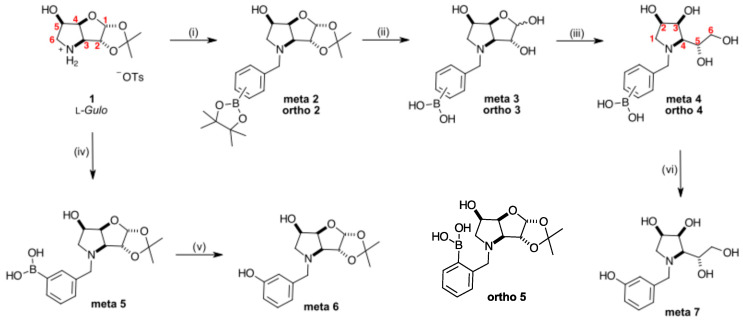
Reaction conditions by synthetic steps (i) **1** (1.1 eq), 3-/2-bromomethylphenylboronic acid pinacol ester (1.0 eq), K_2_CO_3_ (1.0 eq), DMF, 100 °C, 19 h, 72%/71% (respectively). (ii) For **meta 3**: **meta 2**, HCl (aq 1 M), 55 °C, 4 h, quant.; for **ortho 3**: **ortho 2**, HCl (aq 1 M), 55 °C, 18 h, quant. (iii) For **meta 4**: **meta 3** (1.0 eq), NaBH_4_ (3.9 eq), 50% aq EtOH, r.t., 20 h, 84%; for **ortho 4**: **ortho 3** (1.0 eq), NaBH_4_ (2.4 eq), 50% aq EtOH, r.t., 1 h, quant.; (iv) **1** (1.1 eq), 3-bromomethylphenylboronic acid (1.0 eq), K_2_CO_3_ (1.0 eq), DMF, 100 °C, 19 h, 98%; (v) slow oxidation; (vi) **meta 4**, prep t.l.c. (toluene/acetone/acetic acid, 10:15:1), quant.

Divergent intermediate **1** is available via a four-step synthetic protocol [35], starting from reagent 1,2:5,6-di-*O*-isopropylidene-α-d-allofuranose (Scheme 1).

*N*-alkylation, steps (i) and (iv): This reaction (i) required optimisation as initial attempts employing a modified method [36] successfully employed with 6-membered ring systems, was found to be insufficient to drive to completion the reaction of the 5-membered ring. After a thorough investigation of the relevant chemical literature, several strategies can be employed, including S_N_2 [37,38] and reductive amination [39,40,41] on protected [37,39] and unprotected [36,42] iminosugars.

Several strategies were trialled, including a 31 h reaction time at a temperature of 50 °C, after which product formation was confirmed. Acetone was used as reaction solvent in another strategy, requiring a longer reaction time and increased equivalents of potassium carbonate and benzylating agent 3-/2-bromomethylphenylboronic acid pinacol ester. This was likely due to the low solubility of potassium carbonate in this solvent system.

Additional *N*-alkylation strategies trialled, involved one-pot global deprotection of the crude *N*-alkylated product (sequence: steps (i) and (ii)), or global deprotection of divergent iminosugar **1** followed by *N*-alkylation (sequence: steps (ii), (iii) and (i), or steps (ii), (i) and (iii). Neither of these strategies resulted in simplified purification protocols.

The optimised method employs a slight excess of the iminosugar starting material **1** (1.1 eq) compared to the base (K_2_CO_3_) and benzylating agent (2-/3-bromomethylphenyl boronic acid pinacol esters), both utilised in 1.0 equivalent masses. DMF was found to be the best solvent, using a concentration of 10 mg of iminosugar **1**/mL DMF. The resultant crude was dissolved in chloroform and washed with NaOH (aq. 0.1 M), requiring no flash-column purification. This method resulted in high purity products in yields of 72% and 71%, respectively, for **meta 2**, **ortho 2** and **meta 5**.

Purification of boron-bearing organic molecules: Initially, purifications were carried out via flash chromatography (e.g., silica gel) and resin columns (e.g., DOWEX). However, organic boron esters and boronic acids are notoriously difficult to purify due to the Lewis acidity of the B atom and the chemical instability of organic B groups [4]. Purification strategies required optimisation. In our experience, the presence of boronic acid and boronate ester groups greatly widens the range of solvent solubility of molecules they are bonded to. Inevitably, during partitions, several desired intermediates/products tend to travel to both aqueous and organic layers, or to both organic layers (if the partition is carried out solely with organic solvents). Crystallisation, recrystallisations and judicious utilisation of partitioning strategies are powerful tools to maximise yields and minimise chemical instability of boron-bearing molecules.

Global deprotection, step (ii): This was effected by employing HCl (aq. 1 M) at 55 °C to give access to the corresponding lactols **meta 3** and **ortho 3**. Access to **meta 3** required a 4 h reaction time, whereas 18 h were needed for **ortho 3**. Pinacol removal proved difficult and a minor fraction permained.

Reduction, step (iii): The final borylated iminosugars were afforded by a sodium borohydride reduction in lactols **ortho/meta 3**. The optimised reaction conditions were a 1 h reaction time at room temperature with NaBH_4_ (1.2 eq) added at the start of the reaction and an additional portion (1.2 eq) after 30 min. Quenching with glacial acetic acid, followed by filtration and evaporation to dryness gave the final products.

Oxidation side-reaction, steps (v) and (vi): Phenol **meta 6** arose from slow oxidation of **meta 5** over several months, at room temperature. **meta 7** arose when the reaction mixture of **meta 4** was purified by prop t.l.c. The reaction mixture was concentrated in vacuo and triturated, then filtered with methanol/ethanol (1:1). Prep t.l.c. (toluene/acetone/acetic acid, 10:15:1) resulted in oxidation of **meta 4** to **meta 7**.

The detailed ^11^B-NMR, ^1^H-, ^13^C- and 2D NMR data analysis and spectroscopic method for study of mutarotation and borarotation equilibria are available in these publications [30,32,33].

S_N_2 reaction between **1** and 3-bromomethylphenylboronic acid afforded the pinacol-deprotected **meta 5** in excellent yield. For NMR data and analysis, see [33]. Care must be taken in avoiding loss of product in the aqueous layer during the work-up stage. **meta 6** arises from slow oxidation on the bench or in aqueous solution. **ortho 5** arose when a strongly basic aqueous layer (NaOH, 1 M) was utilised to wash the reaction mixture. For NMR data and analysis, see [33].

### 2.2. Biological Assays

#### 2.2.1. Glycosidase Inhibition—Background

**Figure 1:**
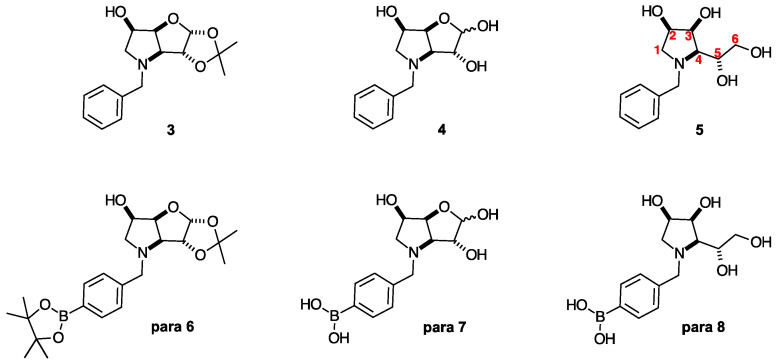
Borylated iminosugars and parent congeners from [30].

Glycosidase inhibition data for N-benzyl-1,4-dideoxy-1,4-imino derivatives are shown in Table 1 and Figure 1.

*N*-Benzyl-1,4-dideoxy-1,4-imino-d-allitol displays selectivity towards α-l-fucosidase (76%) [43], and *N*-benzyl-1,4-dideoxy-1,4-imino-d-galactitol towards α-d-glucosidase (93.2%, IC_50_ = 40.6 μM) [44]. When *N*-benzyl-1,4-dideoxy-1,4-imino-d-mannitol.HCl [45,46,47] is *N*-arylalkylated with short alkyl chains, no glycosidase inhibition is observed [46]. However, lengthening of the *N*-alkyl chain results in increasing inhibition of β-d-glucosidase, β-d-galactosidase and β-d-glucuronidase [45]. Inhibition of α-d-mannosidases is greatest for lysosomal acidic (34%), neutral (44%) and Golgi II (72%) [46], fruit fly lysosomal acidic (IC_50_ = 1.5 × 10^−3^ M) and fruit fly Golgi II (IC_50_ = 6.9 × 10^−4^ M) [47]. *N*-Benzyl-1,4-dideoxy-1,4-imino-d-talitol.HCl poorly inhibited β-d-galactosidase (*E. coli*), α-d-galactosidase (coffee bean) and α-d-mannosidase (Jack bean) [48]. However, the *N*-(2-methylphenyl boronic acid) derivative shows significant inhibition of β-d-galactosidase (*E. coli*) (44–55%), and an increase in inhibition of α-d-galactosidase (coffee bean) (<5%) and α-d-mannosidase (Jack bean) (10%) [48].

Recently, we reported on a family of borylated iminosugars (**para 6**, **para 7** and **para 8**) and their parent compounds (**3**, **4** and **5**) (Figure 1) displaying selective inhibitions (>30%) for β-d-glycosidases (two glucosidases and one galactosidase).

*N*-benzyl-3,6-dideoxy-3,6-imino-d-gulofuranose **4**, *N*-(4-methylphenyl boronic acid)-3,6-dideoxy-3,6-imino-d-gulofuranose **para 7** and *N*-(4-methylphenyl boronic acid)-1,4-dideoxy-1,4-imino-l-gulitol **para 8** selectively inhibited β-d-galactosidase (bovine liver) with, respectively, IC_50_ values of 133 μM (moderate), 218 μM (moderate) and 501 μM (weak). These iminosugars also display an indication of inhibition towards β-d-glucosidases (almond, bovine liver). β-d-glucosidase (EC 3.2.1.21) and β-d-galactosidase (EC 3.2.1.23) are associated with lysosomal storage disorders, such as Gaucher disease [49,50,51] and GM1 gangliosidosis (linked to β-d-glucosidase activity), and Morquio syndrome B (linked to β-d-galactosidase activity) [52]. Rationalisation of inhibition profiles is possible through a systematic postulated framework, such as this one [53].

#### 2.2.2. Glycosidase Inhibitions (Table 2, Scheme 1, Figure 1)

*Controls*: Borocaptate sodium (BSH) and 4-borono-l-phenylalanine (BPA) are clinically used for BNCT. BSH, BPA, and their ^10^B-isotopomers ^10^B-BSH and ^10^B-BPA were utilised as controls. Under the conditions screened, none of them significantly inhibits the glycosidases in the panel at 100 or 1000 μM. Percent inhibitions range from a minimum value of 0 to a maximum value of 19.6.

**Table 2:** Glycosidase inhibition. Percentage inhibition data for controls (BSH, ^10^B-BSH, BPA, ^10^B-BPA), intermediates **ortho 2**, **ortho 3**, **meta 2** and **meta 3**, and target compounds **ortho 4** and **meta 4**, and side products **ortho 5** and **meta 5–7** (drugs) in a panel of 15 glycosidases. IC_50_ values are on green coloured backgrounds (μM): light green denotes weak inhibition, dark green denotes moderate inhibition. NA = not available. Blue background denotes biological activity >30% under conditions screened. In the “Drug” column, on the orange background, the assay concentration legend is as follows: ^a^NI: no inhibition (less than 50% inhibition at 1000 μM), ^b^( ): inhibition % at 1000 μM. In the “Drug” column, on the white background, the assay concentration legend is as follows: ^c^NI: no inhibition (less than 50% inhibition at 100 μM), ^d^( ): inhibition % at 100 μM.

**Drug**	**α-d-** **Glucosidase**				**β-d-** **Glucosidase**			**α-d-** **Galactosidase**	**β-d-** **Galactosidase**	
	**Rice**	**Yeast**	**Rat Intestinal Maltase**	**Human** **Lysosome**	**Almond**	**Bovine Liver**	**Human** **Lysosome**	**Coffee** **Beans**	**Bovine Liver**	***E. coli***
**BSH**	^a^NI ^b^(0%)	^a^NI ^b^(6.9%)	^a^NI ^b^(0%)	NA	^a^NI ^b^(0%)	^a^NI ^b^(15%)	NA	^a^NI ^b^(12.3%)	^a^NI ^b^(0%)	NA
**^10^B-BSH**	^a^NI ^b^(0%)	^a^NI ^b^(5.6%)	^a^NI ^b^(0%)	NA	^a^NI ^b^(0%)	^a^NI ^b^(11.9%)	NA	^a^NI ^b^(3.9%)	^a^NI ^b^(22.3%)	NA
**BPA**	^c^NI ^d^(0%)	^c^NI ^d^(0%)	^c^NI ^d^(0%)	NA	^c^NI ^d^(0%)	^c^NI ^d^(0%)	NA	^c^NI ^d^(4.3%)	^c^NI ^d^(10.9%)	NA
**^10^B-BPA**	^c^NI ^d^(0%)	^c^NI ^d^(0%)	^c^NI ^d^(0%)	NA	^c^NI ^d^(0%)	^c^NI ^d^(14.2%)	NA	^c^NI ^d^(1.2%)	^c^NI ^d^(0%)	NA
**ortho 2**	^c^NI ^d^(0%)	^c^NI ^d^(1%)	^c^NI ^d^(15.5%)	^c^NI ^d^(3.3%)	^c^NI ^d^(35%)	^c^NI ^d^(4.9%)	^c^NI ^d^(5.6%)	^c^NI ^d^(0%)	**362**	^c^NI ^d^(8.8%)
**ortho 2**	^a^NI ^b^(0%)	^a^NI ^b^(0%)	^a^NI ^b^(0%)	NA	^a^NI ^b^(7.2%)	^a^NI ^b^(19.7%)	NA	^a^NI ^b^(2.9%)	^a^NI ^b^(21.8%)	NA
**ortho 3**	^c^NI ^d^(0%)	^c^NI ^d^(2.9%)	^c^NI ^d^(0%)	^c^NI ^d^(0.3%)	^c^NI ^d^(13.7%)	^c^NI ^d^(0%)	^c^NI ^d^(0%)	^c^NI ^d^(0%)	**617**	^c^NI ^d^(4.4%)
**ortho 3**	^a^NI ^b^(0%)	^a^NI ^b^(1%)	^a^NI ^b^(0.7%)	NA	^a^NI ^b^(38.8%)	**710**	NA	^a^NI ^b^(0%)	**141**	NA
**ortho 4**	^c^NI ^d^(0%)	^c^NI ^d^(0%)	^c^NI ^d^(0%)	^c^NI ^d^(2.2%)	^c^NI ^d^(2.6%)	^c^NI ^d^(3.3%)	^c^NI ^d^(0%)	^c^NI ^d^(0%)	^c^NI ^d^(3.3%)	^c^NI ^d^(14%)
**ortho 4**	**622**	**784**	^a^NI ^b^(29.4%)	NA	^a^NI ^b^(40.1%)	^a^NI ^b^(25.7%)	NA	^a^NI ^b^(2.7%)	**533**	NA
**ortho 5**	^c^NI ^d^(6.7%)	^c^NI ^d^(0%)	^c^NI ^d^(0%)	^c^NI ^d^(0%)	^c^NI ^d^(2.7%)	^c^NI ^d^(13.4%)	^c^NI ^d^(3.5%)	^c^NI ^d^(8.5%)	^c^NI ^d^(6.1%)	^c^NI ^d^(0%)
**meta 2**	^c^NI ^d^(0%)	^c^NI ^d^(8.8%)	^c^NI ^d^(5.8%)	^c^NI ^d^(3.9%)	^c^NI ^d^(6.8%)	^c^NI ^d^(0%)	^c^NI ^d^(0%)	^c^NI ^d^(0%)	^c^NI ^d^(2.2%)	^c^NI ^d^(0%)
**meta 2**	^a^NI ^b^(2.9%)	^a^NI ^b^(0%)	^a^NI ^b^(12.4%)	NA	^a^NI ^b^(12.6%)	^a^NI ^b^(32.7%)	NA	^a^NI ^b^(0%)	^a^NI ^b^(41.8%)	NA
**meta 3**	^c^NI ^d^(0%)	^c^NI (1.4%)	^c^NI ^d^(12.3%)	^c^NI ^d^(3.5%)	^c^NI ^d^(24.8%)	^c^NI ^d^(8.2%)	^c^NI ^d^(6.6%)	^c^NI ^d^(0%)	**546**	^c^NI ^d^(0.2%)
**meta 3**	^a^NI ^b^(5.9%)	^a^NI ^b^(37%)	^a^NI ^b^(2.0%)	NA	^a^NI ^b^(45.8%)	**633**	NA	^a^NI ^b^(0.7%)	**116**	NA
**meta 4**	^c^NI ^d^(8%)	^c^NI ^d^(8.1%)	^c^NI ^d^(22.8%)	^c^NI ^d^(7.2%)	^c^NI ^d^(0.6%)	^c^NI ^d^(0%)	^c^NI ^d^(13.6%)	^c^NI ^d^(1.1%)	^c^NI ^d^(24.6%)	^c^NI ^d^(4.9%)
**meta 4**	**106**	^a^NI ^b^(4.9%)	**588**	NA	^a^NI ^b^(43%)	^a^NI ^b^(37.9%)	NA	^a^NI ^b^(3.7%)	**322**	NA
**meta 5**	^c^NI ^d^(0%)	^c^NI ^d^(7.8%)	^c^NI ^d^(0%)	^c^NI ^d^(0.3%)	^c^NI ^d^(0%)	^c^NI ^d^(23%)	^c^NI ^d^(0%)	^c^NI ^d^(1.5%)	^c^NI ^d^(0%)	^c^NI ^d^(0%)
**meta 6**	^c^NI ^d^(0%)	^c^NI ^d^(0%)	^c^NI ^d^(4.7%)	^c^NI ^d^(3.3%)	^c^NI ^d^(6.3%)	^c^NI ^d^(15.8%)	^c^NI ^d^(0%)	^c^NI ^d^(0%)	^c^NI ^d^(15.5%)	^c^NI ^d^(0.8%)
**meta 7**	^c^NI ^d^(0%)	^c^NI ^d^(3.4%)	^c^NI ^d^(0%)	^c^NI ^d^(0%)	^c^NI ^d^(0%)	^c^NI ^d^(0%)	^c^NI ^d^(9.9%)	^c^NI ^d^(1.8%)	^c^NI ^d^(17.5%)	^c^NI ^d^(0%)
**meta 7**	^a^NI ^b^(5.0%)	^a^NI ^b^(4.3%)	^a^NI ^b^(7.2%)	NA	^a^NI ^b^(9.6%)	^a^NI ^b^(13.6%)	NA	^a^NI ^b^(1.7%)	^a^NI ^b^(8.1%)	NA
**Drug**	**α-d-** **Mannosidase**	**β-d-** **Mannosidase**	**α-l-** **Rhamnosidase**	**α-l-** **Fucosidase**	**β-d-** **Glucuronidase**		**Trehalase**	**β-d-** **Glucanase**	**Amyloglucosidase**	
	**Jack Bean**	**Snail**	***P. decumbens***	**Bovine** **Kidney**	***E. coli***	**Bovine Liver**	**Porcine** **Kidney**	***T.*** ***Longibrachiatum***	***A. niger***	
**BSH**	^a^NI ^b^(7.9%)	^a^NI ^b^(19.2%)	^a^NI ^b^(0.2%)	^a^NI ^b^(0%)	^a^NI ^b^(6%)	^a^NI ^b^(19.6%)	^a^NI ^b^(4.2%)	NA	^a^NI ^b^(0%)	
**^10^B-BSH**	^a^NI ^b^(7.7%)	^a^NI ^b^(15%)	^a^NI ^b^(0%)	^a^NI ^b^(6.5%)	^a^NI ^b^(3.2%)	^a^NI ^b^(12.5%)	^a^NI ^b^(2.3%)	NA	^a^NI ^b^(0%)	
**BPA**	^c^NI ^d^(1.1%)	^c^NI ^d^(2.1%)	^c^NI ^d^(0%)	^c^NI ^d^(0%)	^c^NI ^d^(0.5%)	^c^NI ^d^(7.4%)	^c^NI ^d^(0%)	NA	^c^NI ^d^(0%)	
**^10^** **B-BPA**	^c^NI ^d^(0%)	^c^NI ^d^(0.3%)	^c^NI ^d^(0%)	^c^NI ^d^(0%)	^c^NI ^d^(2.4%)	^c^NI ^d^(0%)	^c^NI ^d^(0%)	NA	^c^NI ^d^(0%)	
**ortho 2**	^c^NI ^d^(9.4%)	^c^NI ^d^(0%)	^c^NI ^d^(1.5%)	NA	^c^NI ^d^(4.1%)	^c^NI ^d^(2.2%)	^c^NI ^d^(5.6%)	^c^NI ^d^(21%)	^c^NI ^d^(0%)	
**ortho 2**	^a^NI ^b^(1.3%)	^a^NI ^b^(7.8%)	^a^NI ^b^(2.0%)	^a^NI ^b^(7.8%)	^a^NI ^b^(5.2%)	^a^NI ^b^(8.4%)	^a^NI ^b^(1.8%)	NA	^a^NI ^b^(3.2%)	
**ortho 3**	^c^NI ^d^(3.8%)	^c^NI ^d^(0%)	^c^NI ^d^(0%)	NA	^c^NI ^d^(0%)	^c^NI ^d^(2.9%)	^c^NI ^d^(0%)	^c^NI ^d^(0%)	^c^NI ^d^(0%)	
**ortho 3**	^a^NI ^b^(0%)	^a^NI ^b^(0%)	^a^NI ^b^(3.2%)	^a^NI ^b^(2.1%)	^a^NI ^b^(8.4%)	^a^NI ^b^(6.6%)	^a^NI ^b^(0%)	NA	^a^NI ^b^(2.1%)	
**ortho 4**	^c^NI ^d^(5.6%)	^c^NI ^d^(5.3%)	^c^NI ^d^(4.9%)	NA	^c^NI ^d^(0%)	^c^NI ^d^(0%)	^c^NI ^d^(0%)	^c^NI ^d^(0%)	^c^NI ^d^(0%)	
**ortho 4**	^a^NI ^b^(0.4%)	^a^NI ^b^(2.3%)	^a^NI ^b^(9.5%)	^a^NI ^b^(1.3%)	^a^NI ^b^(31.8%)	^a^NI ^b^(0.9%)	^a^NI ^b^(0%)	NA	^a^NI ^b^(0%)	
**ortho 5**	^c^NI ^d^(12.9%)	^c^NI ^d^(7.0%)	^c^NI ^d^(0%)	NA	^c^NI ^d^(1.2%)	^c^NI ^d^(0%)	^c^NI ^d^(4.9%)	^c^NI ^d^(5.2%)	^c^NI ^d^(4.9%)	
**meta 2**	^c^NI ^d^(7.5%)	^c^NI ^d^(8.4%)	^c^NI ^d^(1.5%)	NA	^c^NI ^d^(13.7%)	^c^NI ^d^(1.4%)	^c^NI ^d^(0%)	^c^NI ^d^(3.9%)	^c^NI ^d^(1.4%)	
**meta 2**	^a^NI ^b^(0%)	^a^NI ^b^(3.7%)	^a^NI ^b^(0%)	^a^NI ^b^(3.8%)	^a^NI ^b^(5.6%)	^a^NI ^b^(10.7%)	^a^NI ^b^(5.8%)	NA	^a^NI ^b^(0%)	
**meta 3**	^c^NI ^d^(0%)	^c^NI ^d^(0%)	^c^NI ^d^(1.2%)	NA	^c^NI ^d^(16.2%)	^c^NI ^d^(1.2%)	^c^NI ^d^(20.9%)	^c^NI ^d^(0%)	^c^NI ^d^(24.6%)	
**meta 3**	^a^NI ^b^(0%)	^a^NI ^b^(0%)	^a^NI ^b^(3.2%)	^a^NI ^b^(3.8%)	^a^NI ^b^(36.9%)	^a^NI ^b^(7.2%)	^a^NI ^b^(0%)	NA	^a^NI ^b^(1.5%)	
**meta 4**	^c^NI ^d^(0%)	^c^NI ^d^(0%)	^c^NI ^d^(8.3%)	NA	^c^NI ^d^(3.5%)	^c^NI ^d^(0%)	^c^NI ^d^(0.8%)	^c^NI ^d^(1.2%)	^c^NI ^d^(6.6%)	
**meta 4**	^a^NI ^b^(0%)	^a^NI ^b^(0%)	^a^NI ^b^(14.7%)	^a^NI ^b^(3.0%)	^a^NI ^b^(20.6%)	^a^NI ^b^(14.7%)	^a^NI ^b^(3.4%)	NA	^a^NI ^b^(3.6%)	
**meta 5**	^c^NI ^d^(6.3%)	^c^NI ^d^(2.3%)	^c^NI ^d^(0.6%)	NA	^c^NI ^d^(4.1%)	^c^NI ^d^(2.9%)	^c^NI ^d^(0%)	^c^NI ^d^(0%)	^c^NI ^d^(0%)	
**meta 6**	^c^NI ^d^(9.5%)	^c^NI ^d^(0.6%)	^c^NI ^d^(6.5%)	NA	^c^NI ^d^(0%)	^c^NI ^d^(1.6%)	^c^NI ^d^(0%)	^c^NI ^d^(3.1%)	^c^NI ^d^(4.8%)	
**meta 7**	^c^NI ^d^(0%)	^c^NI ^d^(0%)	^c^NI ^d^(3.8%)	NA	^c^NI ^d^(0.5%)	^c^NI ^d^(1.2%)	^c^NI ^d^(0%)	^c^NI ^d^(0%)	^c^NI ^d^(5.1%)	
**meta 7**	^a^NI ^b^(0%)	^a^NI ^b^(0%)	^a^NI ^b^(2.1%)	^a^NI ^b^(2.1%)	^a^NI ^b^(7.5%)	^a^NI ^b^(0%)	^a^NI ^b^(1.9%)	NA	^a^NI ^b^(2.1%)	

*Glycosidase Inhibitions*: For quantitative comparison of glycosidase inhibition, these ranges are used: weak (IC_50_ > 250 μm), moderate (IC_50_ = 100–249 μm), good (IC_50_ = 10–99 μm), potent (IC_50_ = 0.1–9 μm) and very potent (IC_50_ < 0.1 μm) inhibition [53]. Table 2 is packed with glycosidase inhibition and structure–activity relationships data. Table 2 needs to be analysed in conjunction with glycosidase inhibition data in [30].

Top-down overview of the inhibition data shows two stark features dominating Table 2: the selectivity towards β-d-galactosidase (bovine liver), and the emerging inhibition towards β-d-glucosidases (almond, bovine liver). The top inhibitions are for **meta 4** towards α-d-glucosidase (rice) with an IC_50_ = 106 μm, for **meta 3** towards β-d-galactosidase (bovine liver) with an IC_50_ = 116 μm, and for **ortho 3** towards β-d-galactosidase (bovine liver) with an IC_50_ = 141 μm.

*Comparison of protected intermediates* **3**, **para 6** [30], **meta 2** *and* **ortho 2**. These protected intermediates would be expected to have minimal or no activity. In this context **ortho 2** fares remarkably well towards β-d-galactosidase (bovine liver) with an IC_50_ = 362 μM and an emerging inhibition profile for β-d-glucosidases (almond, bovine liver) for **5** (31.9%), **ortho 2** (35%) and **meta 2** (32.7%).

*Comparison of deprotected lactol intermediates* **4**, **para 7** [30], **meta 3** *and* **ortho 3**. The inhibitory capability of these intermediates increases significantly compared to their protected precursors. The boronic acid is more accessible to interact with glycosidases. The lactol group tends to display poor metabolic stability [54,55,56,57]. In this context **ortho 3** fares remarkably well towards β-d-galactosidase (bovine liver) with an IC_50_ = 617 μM and **meta 3** inhibits the same enzyme with an IC_50_ = 546 μM. At 1000 μM concentration, selectivity is retained towards β-d-galactosidase (bovine liver). An emerging inhibition profile for β-d-glucosidases (almond, bovine liver) can be seen for **para 7** (43.6% and 38.8%, respectively) and **ortho 3** (38.8%). An emerging inhibition of β-d-glucuronidase (*E. coli*) can be seen for **meta 3** (36.9%). **meta 3** and **ortho 3** inhibit β-d-glucosidases (bovine liver) with IC_50_s = 633 and 710 μM, respectively. Moderate IC_50_s are displayed by all compounds towards β-d-galactosidase (bovine liver) with IC_50_s = 133 μM (for **4**), 218 μM (for **para 7**), 116 μM (for **meta 3**) and 141 μM (for **ortho 3**). This indicates the iminosugar absolute stereochemistry is important in selecting the enzyme.

*Comparison of target compounds* **5**, **para 8** [30], **meta 4**, **meta 7** *and* **ortho 4**. Comparison of **meta 7** with **meta 4** shows complete abrogation of inhibition when the boronic acid group is replaced with an OH group. This indicates establishment of favourable interactions between the boronic acid group with several glycosidases: β-d-glucosidases (almond) 43%, (bovine liver) 37.9%, α-d-glucosidase (rat intestinal maltase) IC_50_ = 588 μm, β-d-galactosidase (bovine liver) IC_50_ = 322 μm, culminating with inhibition of α-d-glucosidase (rice) IC_50_ = 106 μm. Comparison of **meta 4** and **ortho 4** shows they have similar inhibition profiles towards α-d-glucosidase (rice), β-d-glucosidase (almond), and β-d-galactosidase (bovine liver) albeit with reduced potency for **ortho 4**. **ortho 4** additionally weakly inhibits α-d-glucosidase (yeast) with an IC_50_ = 784 μm. Comparison to their non-borylated analogue **5** [30] shows biological activity is abrogated, pointing to a preponderant role for the boronic acid group in its interactions with glycosidases.

Comparison to their **para 8** congener shows inhibition of β-d-galactosidase (bovine liver) IC_50_ = 501 μm, again indicating a role for the boronic acid in interactions with the same family of glycosidases, but further reduced.

*Activity of side-products* **meta 5**, **meta 6**, **meta 7** *and* **ortho 5**. The presence of the acetonide protecting group abrogates inhibitory activity in **meta 5**, **meta 6 and ortho 5**.

Lack of boronic acid group in **meta 7** also results in abrogation of biological activity. These data confirm that both the iminosugar absolute stereochemical configuration and the presence of the boronic acid group are both important to activity.

For comparison, other structurally related glycosidase inhibitors include the natural product 1-DNJ, which is a good inhibitor of α-galactosidase (green coffee beans, IC_50_ 16 µM), α-glucosidase (*Saccharomyces cerevisiae*, IC_50_ 35 µM), β-glucosidase (almond, IC_50_ 71 µM) [58], and amyloglucosidase (IC_50_ 2.1 µM) [59]. These are comparable potencies to the best inhibitors presented here. However, 1-DNJ lacks specificity of inhibition. *N*-Butyl-DNJ, marketed as Zavesca^®^ (miglustat) for the management of Gaucher Disease inhibits glucocerebrosidase with an IC_50_ 5–50 µM [60]. It also shows inhibition of α-glucosidases (endoplasmatic reticulum I and II, IC_50_ 0.68 µM and 10.8 µM, respectively) [61]. Basen^®^ (voglibose), a clinical agent for the management of diabetes, inhibits α-glucosidases (rat maltase, rat isomaltase and rat sucrase with IC_50_ 0.11, 0.16 and 0.07 µM, respectively).

#### 2.2.3. Glycosidase Inhibitions (Table 3, Scheme 1, Figure 1)

*Controls*: Under the assay conditions, BSH and its ^10^B-BSH inhibited several glycosidases (31.6–65.9%). BPA and ^10^B-BPA inhibitions were all <30%.

**Table 3:** Glycosidase inhibition. Percentage inhibition data for controls (BSH, ^10^B-BSH, BPA, ^10^B-BPA), and borylated intermediates, target compounds and side-products (drugs) in a panel of five-eight glycosidases. NA = not available. The pale blue background denotes inhibitory activity 30–60%, and the blue background inhibitory activity >60%, under the conditions screened. Sample preparation: 1 mL of water was added to 1 mg solid. In bold are highlighted the % inhibitions >13 as significant.

Drug	Sample Appearance	α-d-Glucosidase			β-d-Glucosidase	α-d-Mannosidase	*N*-Acetyl-β-d-glucosaminidase	*N*-Acetyl-β-d-Hexosaminidase	β-d-Glucuronidase
		Yeast	Bacillus	Rat Intestine	Almond	Jack Bean	Bovine Kidney	Rat Intestine	Bovine Liver
**BSH**	In solution	59	48.1	NA	47.3	−18.7	37.1	NA	31.6
**^10^B-BSH**	In solution	65.9	53	NA	49.9	−16.8	40.9	NA	44.1
**BPA**	Some in solution with undissolved sediment	2.9	19.9	NA	3.9	0.6	6.7	NA	−0.7
**^10^B-BPA**		3.4	19.5	NA	3	−0.7	6.3	NA	−1
**ortho 2**	In solution	−2	NA	−6.7	**73.3**	NA	NA	**13.8**	−1.8
**ortho 3**	In solution	4.1	NA	−1.4	**68.6**	NA	NA	**19.2**	1.3
**ortho 4**	In solution	−6.7	NA	**17.4**	**49.8**	NA	NA	−0.2	2.2
**ortho 5**	In solution	8.7/11	NA	4.2	8.7	NA	NA	5.2	1.4
**meta 2**	In solution	9.3/7.9	NA	**16.8**	**13.2**	NA	NA	11.5	9.9
**meta 3**	In solution	−16.7	NA	12.1/8.9	**61.7**	NA	NA	8.7	**13.3**
**meta 4**	In solution	**23.5**	NA	**41.1**	**47.9**	NA	NA	−2.2	−2.7
**meta 5**	In solution	**14.8/13.7**	NA	11.6	7.7	NA	NA	5	−0.9
**meta 6**	In solution	6.6	NA	4.8	0.7	NA	NA	−0.7	3.6
**meta 7**	In solution	**19.5**	NA	−10.7	2.9	NA	NA	−2.5	−3

*Glycosidase Inhibitions*: Table 3 needs to be analysed in conjunction with glycosidase inhibition data in [30]. The predominant feature in Table 3 is represented by the inhibition of β-d-glucosidase (almond) and an emerging inhibition towards α-d-glucosidases.

Inhibition of β-d-glucosidase (almond) was selective and strong for **para 7** (68.1%), **meta 3** (61.7%), **ortho 2** (73.3%) and **ortho 3** (68.6%). β-d-glucosidase (almond) is also inhibited by **meta 4** (47.9%) and **ortho 4** (49.8%). **ortho 3** also inhibits *N*-acetyl-β-d-hexosaminidase (rat intestine) (19.2%), **meta 4** shows activity also against α-d-glucosidase (rat intestine) (41.1%) and α-d-glucosidase (yeast) (23.5%), **meta 7** inhibits weakly α-d-glucosidase (yeast) (19.5%), **ortho 4** shows an emerging inhibition of α-d-glucosidase (rat intestine) (17.4%).

The strongest inhibition was seen for **para 7** which also inhibits β-d-glucosidase (almond) in a significant amount (68.1%, strong inhibition), mirroring the measurement in Table 2.

#### 2.2.4. Cancer Screening (Table 4, Scheme 1, Figure 1)

The assays are the dose screen to ascertain cell growth inhibition in response to 25 µM of drug (on the blue background) and the dose response (GI_50_) (on the green background). For a discussion of BNCT as a radiotherapeutic modality, a rationalisation is provided here [27].

**Table 4:** Cancer screening. On the blue background DOSE SCREEN: percentage (%) cell growth inhibition in response to 25 µM of drug (the higher the value, the greater the growth inhibition) and inhibition value ranges: 35–59% (green), 10–34% (blue) and 0–9% (black). On the green background DOSE RESPONSE: GI_50_ = concentration (µM) that inhibits cell growth by 50% (the lower the value, the greater the growth inhibition). In bold are highlighted the values of the most potent inhibition. * n = 3–4, otherwise n = 2.

	Carcinomas					Normal
Drug	HT29 Colon	A2780 Ovarian	H460 Lung	A431 Skin	MIA-Pa-Ca2 Pancreatic	MCF10A Breast
**BSH**	* 3 ± 2	* 2 ± 5	* 8 ± 2	* <0	* 2 ± 6	* 8 ± 3
**^10^B-BSH**	* 5 ± 1	* 5 ± 4	* 4 ± 2	* <0	* 2 ± 4	* 13 ± 4
**BPA**	* 14 ± 0	* 4 ± 1	* 7 ± 8	* 4 ± 6	* 3 ± 3	* 4 ± 1
**^10^B-BPA**	* 15 ± 4	* 8 ± 4	* 8 ± 5	* 4 ± 4	* 11 ± 3	* <0
**ortho 2**	7 ± 0	7 ± 2	11 ± 4	10 ± 5	9 ± 7	9 ± 4
	>50	>50	>50	>50	>50	>50
**ortho 3**	<0	17 ± 2	2 ± 1	3 ± 1	5 ± 5	9 ± 7
	>50	>50	>50	>50	>50	>50
**ortho 4**	<0	16 ± 8	8 ± 2	17 ± 2	7 ± 2	8 ± 4
	>50	>50	>50	>50	>50	>50
**ortho 5**	4 ± 4	19 ± 2	2 ± 2	9 ± 4	12 ± 3	7 ± 6
	>50	>50	>50	>50	>50	>50
**meta 2**	1 ± 10	25 ± 2	5 ± 2	8 ± 6	19 ± 8	6 ± 4
	>50	>50	>50	>50	>50	>50
**meta 3**	<0	18 ± 1	2 ± 2	3 ± 0	17 ± 4	6 ± 4
	>50	>50	>50	>50	>50	>50
**meta 4**	<0	18 ± 5	7 ± 1	6 ± 1	16 ± 5	14 ± 13
	>50	>50	>50	>50	>50	>50
**meta 5**	<0	32 ± 7	10 ± 2	8 ± 8	16 ± 1	7 ± 4
	>50	>50	>50	>50	>50	>50
**meta 6**	4 ± 3	23 ± 6	6 ± 3	4 ± 9	12 ± 4	4 ± 2
	>50	>50	>50	>50	>50	>50
**meta 7**	29 ± 1	**48 ± 7**	**37 ± 3**	**56 ± 6**	**39 ± 2**	31 ± 16
	50 ± 0.0	30 ± 20	47 ± 7.0	17 ± 8.5	51 ± 1.5	43 ± 17

*Controls*: The controls do not inhibit cell growth at 25 µM with cell growth inhibitions ranging from < 0% to 19% in our panel of five cancer cell lines and a normal cell line. Only six out of 24 entries for the controls were 10%—or greater—percent inhibitions. Three entries have values of 0% or < 0%. The remaining inhibitions ranged between 0.01% and 9.99%.

*Cancer Screening*: Table 4 needs to be analysed in conjunction with cancer screening data in [30]. From a structure–activity relationship perspective, the A2780 ovarian carcinoma cell line is inhibited by all compounds apart from **ortho 2**. A second main feature is inhibition of the MIA-Pa-Ca2 pancreatic carcinoma cell line.

Overall, weak/moderate cell growth inhibitions are displayed with most between 10% and 32% (blue), and between 39% and 56% for **meta 7**, which inhibits all cancer cell lines, including the MCF10A breast (normal) cell line. GI_50_s for **meta 7** were also significant with values ranging from 51 µM to 17 µM. **meta 7** also inhibits the MCF10A breast (normal) cell line with a GI_50_ = 43 µM.

Addition of the boron groups tempers the potency of inhibition. More important than the potency of inhibition and dose-response capabilities, is the capability for switch on/switch off activation of these borylated drugs under BNCT radiotherapeutic conditions. This is where their real therapeutic potential resides. This is when they become destructive to the cancer molecules, provided they can selectively accumulate in them versus healthy cells. We have demonstrated in earlier work that this is possible [29,34,62].

## 3. Materials and Methods

### 3.1. Glycosidase Inhibition for Table 2

The enzymes α-d-glucosidase (yeast), β-d-glucosidases (almond, bovine liver), α-d-galactosidase (coffee beans), β-d-galactosidase (bovine liver), α-d-mannosidase (Jack bean), β-d-mannosidase (snail), α-l-rhamnosidase (*P. decumbens*), α-l-fucosidase (bovine kidney), trehalase (porcine kidney), β-d-glucuronidases (*E. coli*, bovine liver), amyloglucosidase (*A. niger*), para-nitrophenyl glycosides, and various disaccharides were purchased from Sigma-Aldrich Co (St. Louis, MO, USA).

Brush border membranes were prepared from the rat small intestine according to the method of Kessler et al. [63], and were assayed at pH 6.8 for rat intestinal maltase using maltose. For rat intestinal maltase, porcine kidney trehalase, and *A. niger* amyloglucosidase activities, the reaction mixture contained maltose (25 μM) and the appropriate amount of enzyme, and the incubations were performed for 10–30 min at 37 °C. The reaction was stopped by heating at 100 °C for 3 min. After centrifugation (600 g; 10 min), the resulting reaction mixture was added to the Glucose CII-test Wako (Wako Pure Chemical Ind., Osaka, Japan). The absorbance at 505 nm was measured to determine the amount of the released d-glucose. Other glycosidase activities were determined using an appropriate *para*-nitrophenyl glycoside as substrate at the optimum pH of each enzyme. The reaction mixture contained the substrate (2 μM) and the appropriate amount of enzyme. The reaction was stopped by the addition of Na_2_CO_3_ (400 μM). The released para-nitrophenol was measured spectrometrically at 400 nm. All reactions were run in methanol.

### 3.2. Glycosidase Inhibition for Table 3

All enzymes and *para*-nitrophenyl substrates were purchased from Sigma. Enzymes were assayed at 27 °C in citric acid (0.1 M)/disodium hydrogen phosphate (0.2 M) buffers at the optimum pH for the enzyme. The incubation mixture consisted of 10 mL enzyme solution, 10 mL of 1 mg/mL aqueous solution of extract and 50 mL of the appropriate 5 μM *para*-nitrophenyl substrate made up in buffer at the optimum pH for the enzyme. The reactions were stopped by addition of 70 mL 0.4 M glycine (pH 10.4) during the exponential phase of the reaction, which had been determined at the beginning using uninhibited assays in which water replaced the inhibitor. Final absorbances were read at 405 nm using a Versamax microplate reader (Molecular Devices, Wokingham, UK). Assays were carried out in triplicate, and the values given are means of the three replicates per assay. All reactions were run in water.

### 3.3. Cancer Screening

For Table 4, all test agents were prepared as stock solutions (20 μM) in dimethyl sulfoxide (DMSO) and stored at −20 °C. Cell lines used in the study included HT29 (colorectal carcinoma), A2780 (ovarian carcinoma), H460 (lung carcinoma), A431 (skin carcinoma), MiaPaCa-2 (pancreatic carcinoma), and SMA560 (spontaneous murine astrocytoma), together with MCF10A (non-tumour derived normal breast cell line). Cell line sources: the HT29, H460, and MiaPaCa-2, together with MCF10A were purchased from ATCC. The A2780 and A431 were purchased from the European Collection of Cell Culture (ECACC). All cell lines were incubated in a humidified atmosphere 5% CO_2_ at 37 °C. The cancer cell lines were maintained in Dulbecco’s modified Eagle’s medium (DMEM; Sigma, Truganina, Australia) supplemented with foetal bovine serum (10%), sodium pyruvate (10 μM), penicillin (100 IUmL^−1^), streptomycin (100 µg mL^−1^), and l-glutamine (2 μM).

The non-cancer MCF10A cell line was maintained in DMEM:F12 (1:1) cell culture media, 5% heat inactivated horse serum, supplemented with penicillin (50 IUmL^−1^), streptomycin (50 µg mL^−1^), HEPES (20 μM), l-glutamine (2 μM), epidermal growth factor (20 ng mL^−1^), hydrocortisone (500 ng mL^−1^), cholera toxin (100 ng mL^−1^), and insulin (10 mg mL^−1^).

Growth inhibition was determined by plating cells in duplicate in medium (100 µL) at a density of 2500–4000 cells per well in 96-well plates. On day 0 (24 h after plating), when the cells are in logarithmic growth, medium (100 µL) with or without the test agent was added to each well. After 72 h drug exposure, growth inhibitory effects were evaluated using the MTT (3-(4,5-dimethyltiazol-2-yl)-2,5-diphenyltetrazolium bromide) assay and absorbance read at 540 nm. The percentage growth inhibition was calculated at a fixed concentration of 25 µM, based on the difference between the optical density values on day 0 and those at the end of drug exposure. Each data point is the mean ± the standard error of the mean (SEM) calculated from three replicates which were performed on separate occasions and separate cell line passages.

### 3.4. Numbering System

Spectroscopic data for all compounds are assigned based on a numbering system derived from systematic naming of materials according to IUPAC recommendations on carbohydrate nomenclature [64]. The numbering is given in Scheme 1 by the red numbers and letters on selected structures.

### 3.5. General Chemical Characterisation Methods

Spectra were recorded on a Bruker Ascend^TM^ 400 in deuterated solvent as stated. Chemical shifts (δ) are quoted in ppm and coupling constants (*J*) in Hz. Residual signals from the CDCl_3_ (7.26 ppm in ^1^H-NMR, 77.16 ppm in ^13^C-NMR), deuterated acetone (2.05 ppm in ^1^H-NMR, 29.84 ppm in ^13^C-NMR), deuterated methanol (3.31 ppm in ^1^H-NMR, 49.00 ppm in ^13^C-NMR), deuterated acetic acid (2.04 ppm and 11.65 ppm in ^1^H-NMR, 178.0 ppm and 20.0 ppm in ^13^C-NMR) and deuterium oxide (4.79 ppm in ^1^H-NMR) were used as an internal reference [65]. NMR spectra in the Supplementary Information were produced utilising TopSpin 4.2.0 [66]. The boron hump is visible between ~10 and ~−40 ppm in the ^11^B-NMR spectra.

Infrared (IR) Spectroscopy: IR spectra were obtained on a PerkinElmer Spectrum Two Spectrometer and on a PerkinElmer Spectrum 2 with UATR. Only characteristic peaks are quoted and in units of cm^−1^. Vibrations relating to boron-containing groups were derived and assigned from [67,68,69,70,71,72].

Low Resolution Mass Spectrometry (LRMS) spectra were obtained on an Agilent Technologies 1260 Infinity UPLC system with a 6120 Quadrupole LC/MS in electrospray ionisation (ESI) positive and negative modes. All LCMS methods used a mobile phase A of 100% water with 0.1% formic acid and mobile phase B of 9:1 *v*/*v* ACN/water with 0.1% formic acid.

Optical rotations were carried out on a Jasco P-2000 polarimeter with a length of 1.0 dm. Concentrations are quoted in g/100 mL.

### 3.6. Reagents and Solvents

Solvents: Dichloromethane, *N,N*-dimethylformamide (DMF), acetic acid and pyridine were purchased from the Aldrich Chemical Company in sure-seal^TM^ reagent bottles. Reverse osmosis water was used. All other solvents (analytical or HPLC grade) were used as supplied without further purification. The 2-/3-bromomethylphenyl boronic acid pinacol esters were purchased from Boron Molecular. Deuterated solvents were purchased from Cambridge Isotope Laboratories.

Reagents: The reagents were used as provided without further purification, with NMR analysis confirming an acceptable degree of purity and correct structural identity.

Purification via prep t.l.c. were performed on Davisil 40–63-micron silica gel. Thin layer chromatography (t.l.c.) was performed on aluminium sheets coated with 60 F254 silica by Merck and visualised using UVG-11 Compact UV lamp (254 nm) or stained with a solution of ammonium molybdate (12 g) and ceric ammonium molybdate (0.5 g) in concentrated sulfuric acid (15 mL) and distilled water (235 mL) and heated until development.

### 3.7. Chemistry Experimental Method

#### 3.7.1. *N*-(3-Methylphenyl boronic acid pinacol ester)-3,6-dideoxy-3,6-imino-1,2-*O*-isopropylidene-α-d-gulofuranose **meta 2** (Scheme 1, Step (i))

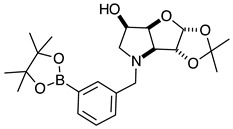



3,6-Dideoxy-3,6-imino-1,2-*O*-isopropylidene-α-d-gulofuranose *p*-toluenesulfonate **1** (194 mg, 0.520 mmol, 1.1 eq) was dissolved and stirred in DMF (20 mL). K_2_CO_3_ (67 mg, 0.487 mmol, 1.0 eq) and 3-bromomethylphenyl boronic acid pinacol ester (145 mg, 0.243 mmol, 1.0 eq) were added portionwise. The reaction was heated to 100˚C and allowed to stir for 19 h. T.l.c. analysis (ethyl acetate/hexane, 1:4) showed presence of one product (R*_f_* 0.34) and consumption of the starting material (R*_f_* 0.00). The solvent was evaporated to dryness, the crude residue gently triturated in chloroform (60 mL) and washed with NaOH (aqueous 0.1 M, 20 mL). The organic layer was evaporated to dryness to give the desired product *N*-(3-methylphenyl boronic acid pinacol ester)-3,6-dideoxy-3,6-imino-1,2-*O*-isopropylidene-α-d-gulofuranose **meta 2** as a brown oil (156 mg, 0.374 mmol, 72%). m/z (HRMS ES^+^): found mass 418.2 [M+H^+^]^+^, required mass 418.2 [M+H^+^]^+^; [α]_289_^19^ 0.11 (c 0.037 in MeOH); ν_max_ (thin film, cm^−1^): 3391 (m, broad, OH), 3056 (w, ArCH), 2981, 2938 (m), 2876, 2805 (w, alkyl CH), 1652 (w), 1608 (w), 1488 (w), 1457 (w), 1430 (w, ArC=C), 1372 (m, B-O), 1356 (s, B-O), 1322 (m, C-O), 1274 (m), 1214 (m, B-O-H bend), 1164 (m, OH bend), 1143 (s), 1069 (s, B-C), 1013 (m), 965 (m), 922 (m), 881, (w, meta-subst), 852 (s), 814 (m, meta-subst), 710 (s), 672 (m, meta-subst), 621 (m), 567 (m), 516 (w), 439 (w), 412 (w). For NMR data and analysis, see [33].

#### 3.7.2. *N*-(3-Methylphenyl boronic acid)-3,6-dideoxy-3,6-imino-d-gulofuranose **meta 3** (Scheme 1, Step (ii))

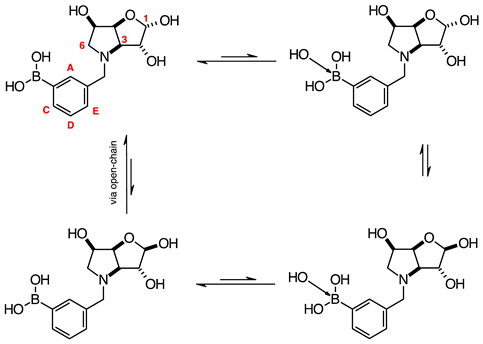



*N*-(3-Methylphenyl boronic acid pinacol ester)-3,6-dideoxy-3,6-imino-1,2-*O*-isopropylidene-α-d-glucofuranose **meta 2** (117 mg, 0.279 mmol) was dissolved in HCl (aqueous 1 M, 12 mL) and allowed to stir at 55˚C for four hours. T.l.c. analysis (ethyl acetate/hexane, 1:4) indicated complete consumption of the starting material (R*_f_* 0.29) and appearance of a single spot (R*_f_* 0.00). The solvent was evaporated to dryness to give product *N*-(3-methylphenyl boronic acid)-3,6-dideoxy-3,6-imino-d-gulofuranose **meta 3** and the free pinacol as a brown oil (quant). m/z (HRMS ES^+^): found mass 296.1 [M+H^+^]^+^, required mass 296.1 [M+H^+^]^+^; [α]_289_^19^ 0.34 (c 0.035 in MeOH); ν_max_ (thin film, cm^−1^): 3318 (s, broad, OH), 2980 (m, with shoulders), 1639 (m, with shoulder), 1610 (m), 1525 (w), 1433 (m, ArC=C), 1359 (s, B-O), 1278 (m, C-O), 1212 (m, B-O-H bend), 1193 (s, OH bend), 1143 (s), 1123 (s), 1109 (s), 1083 (s, B-C), 1035 (s), 1011 (s), 980 (m), 964 (m), 952 (m), 890 (m, meta-subst), 852 (m), 813 (m), 786 (m, meta-subst), 708 (m), 682 (m), 665 (s, meta-subst), 565 (s), 555 (s). For NMR data and analysis, see [33].

#### 3.7.3. *N*-(3-Methylphenyl boronic acid)-1,4-dideoxy-1,4-imino-l-gulitol **meta 4** (Scheme 1, Step (iii))

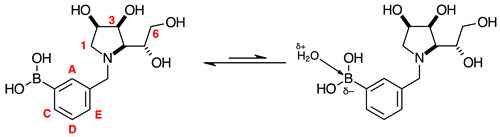



*N*-(3-Methylphenyl boronic acid)-3,6-dideoxy-3,6-imino-d-gulofuranose **meta 3** (61 mg, 0.205 mmol) was dissolved in ethanol (aqueous 50%, 10 mL) prior to the addition of NaBH_4_ (9.4 mg, 0.246 mmol, 1.2 eq) and the reaction was allowed to stir at r.t. After 30 min, an additional portion of NaBH_4_ (11 mg, 0.298 mmol, 1.5 eq) was carefully added and the reaction stirred for one more hour. T.l.c. analysis (toluene/acetone/acetic acid, 5.0:4.5:0.5) showed the reaction had not yet gone to completion, so an additional portion of NaBH_4_ (9 mg, 0.246 mmol, 1.2 eq) was added and stirred at r.t. for 18 h. T.l.c. analysis (toluene/acetone/acetic acid, 5.0:4.5:0.5) showed formation of one product (R*_f_* 0.00) and consumption of the starting material (R*_f_* 0.65). After quenching with glacial acetic acid (a few drops), the mixture was evaporated to dryness to give a crude residue (112 mg). This crude residue (50 mg) was triturated with methanol/ethanol (1:1), filtered and dried to give product *N*-(3-methylphenyl boronic acid)-1,4-dideoxy-1,4-imino-l-gulitol **meta 4** as a brown residue (23 mg, 0.077 mmol, 84%). [α]_289_^19^ −0.09 (c 0.033 in H_2_O); ν_max_ (thin film, cm^−1^): 3330 (s, broad, OH), 2956 (m, with shoulders, alkyl CH), 2737, 2619, 1603 (m, with shoulder), 1593 (m), 1459 (m, ArC=C), 1368 (m, B-O), 1285 (m, C-O), 1213 (m, B-O-H bend), 1171 (m, OH), 1107 (s, B-C), 1036 (s), 1012 (m), 959 (m, with shoulder, meta-subst), 817 (w, ), 792 (w, meta-subst), 756 (w), 703 (w), 686 (w, meta-subst), 568 (w), 533 (w). For NMR data and analysis, see [33].

#### 3.7.4. *N*-(2-Methylphenyl boronic acid pinacol ester)-3,6-dideoxy-3,6-imino-1,2-*O*-isopropylidene-α-d-gulofuranose **ortho 2** (Scheme 1, Step (i))

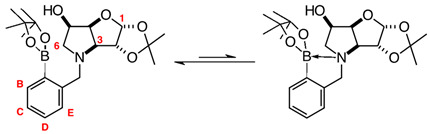



3,6-Dideoxy-3,6-imino-1,2-*O*-isopropylidene-α-d-gulofuranose *p*-toluenesulfonate **1** (100 mg, 0.268 mmol, 1.1 eq) was dissolved and stirred in DMF (10 mL). K_2_CO_3_ (34 mg, 0.243 mmol, 1.0 eq) and 2-bromomethylphenyl boronic acid pinacol ester (72 mg, 0.243 mmol, 1.0 eq) were added portionwise. The reaction was heated to 100˚C and allowed to stir for 19 h. T.l.c. analysis (ethyl acetate/hexane, 1:4) showed presence of one product (R*_f_* 0.35) and consumption of the starting material (R*_f_* 0.00). The solvent was evaporated to dryness and the crude dissolved in chloroform (30 mL) and washed with NaOH (aqueous 0.1 M, 10 mL). The organic layer was evaporated to dryness to give product *N*-(2-methylphenyl boronic acid pinacol ester)-3,6-dideoxy-3,6-imino-1,2-*O*-isopropylidene-α-d-gulofuranose **ortho 2** as a brown oil (79 mg, 0.190 mmol, 71%). m/z (HRMS ES^+^): found mass 418.2 [M+H^+^]^+^, required mass 418.2 [M+H^+^]^+^; [α]_298_^25^ 0.15 (c 0.08 in MeOH); ν_max_ (thin film, cm^−1^): 3505 (broad, w, OH), 3047 (w, ArCH), 2983 (m) 2930 (m) 2868 (w) 2803 (w, alkyl CH), 1723, 1669 (w, ortho-subst, overtones), 1601 (m), 1570 (w), 1492 (m), 1455 (m), 1444 (m, ArC=C), 1381 (s, B-O), 1372 (s, B-N), 1346 (s, B-O), 1313 (s, C-O), 1262 (m), 1216 (s, B-O-H bend), 1163 (s, OH bend), 1144 (s), 1084 (s, B-C), 1066 (s) and 1040 (s, C-N bend and stretch), 1019 (s), 963 (m), 883 (m), 861 (s), 751 (s, ortho-subst), 662 (s), 578 (w), 565 (w), 555 (w), 515 (w), 493 (w). For NMR data and analysis, see [33].

#### 3.7.5. *N*-(2-Methylphenyl boronic acid)-3,6-dideoxy-3,6-imino-d-gulofuranose **ortho 3** (Scheme 1, Step (ii))

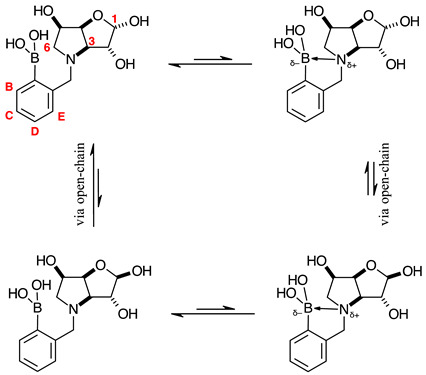



*N*-(2-Methylphenyl boronic acid pinacol ester)-3,6-dideoxy-3,6-imino-1,2-*O*-isopropylidene-α-d-gulofuranose **ortho 2** (69 mg, 0.164 mmol) was dissolved in HCl (aqueous 1 M, 7 mL) and allowed to stir at 55˚C for 18 h. T.l.c. analysis (ethyl acetate/hexane, 1:4) indicated complete consumption of the starting material (R*_f_* 0.29) and appearance of a single spot (R*_f_* 0.0). The solvent was evaporated to dryness to give the desired product *N*-(2-methylphenyl boronic acid)-3,6-dideoxy-3,6-imino-d-gulofuranose **ortho 3** and the remaining free pinacol as a brown residue (quant). m/z (HRMS ES^+^): found mass 296.1 [M+H^+^]^+^, required mass 296.1 [M+H^+^]^+^; [α]_289_^25^ 0.18 (c 0.065 in MeOH); ν_max_ (thin film, cm^−1^): 3329 (broad, s, OH), 3081 (w, ArCH), 2982, 2947 (m, alkyl CH), 2700, 2593, 1717 (w, ortho-subst, overtones), 1639 (w), 1602 (w, sharp), 1573 (w), 1496 (w), 1447 (m, ArC=C), 1352 (s, with shoulder, B-O), 1325 (s, C-O), 1273 (m), 1210 (m, B-O-H bend), 1178 (m, OH bend), 1142 (s), 1124 (s), 1072 (s, B-C), 1044 (s), 981 (m), 964 (m), 858 (m), 767 (m, ortho subst), 702 (m), 658 (m), 633 (m), 566 (m), 556 (m), 512 (m). For NMR data and analysis, see [33].

#### 3.7.6. *N*-(2-Methylphenyl boronic acid)-1,4-dideoxy-1,4-imino-l-gulitol **ortho 4** (Scheme 1, Step (iii))

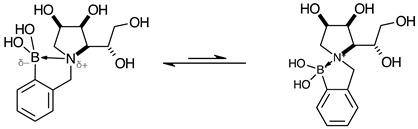



*N*-(2-Methylphenyl boronic acid)-3,6-dideoxy-3,6-imino-d-glucofuranose **ortho 3** (34 mg, 0.114 mmol) was dissolved in ethanol (aqueous 50%, 20 mL) prior to the addition of NaBH_4_ (5.5 mg, 0.137 mmol, 1.2 eq). The reaction was then allowed to stir at room temperature for 30 min. One more portion of NaBH_4_ (6 mg, 0.137 mmol, 1.2 eq) was carefully added and the reaction stirred for an additional 30 min. After quenching with glacial acetic acid (a few drops) and filtered, the solvent was removed to give product *N*-(2-methylphenyl boronic acid)-1,4-dideoxy-1,4-imino-l-gulitol **ortho 4** (quant.) as an off-white residue. [α]_289_^19^ 0.06 (c 0.031 in H_2_O); ν_max_ (thin film, cm^−1^): 3201 (broad s, OH), 2987, 2929 and 2861 (w, alkyl CH), 1738 (w, ortho-subst, overtones), 1654 (sharp, s), 1602 (w, ArC=C), 1414 (w) and 1387 (m, B-O), 1294 (m, C-O), 1253 (m), 1143 (w), 1099 (m, B-C), 1049 and 1028 (m, C-N bend and stretch), 981, 895, 870, 772, 730 (m, ortho-subst), 662, 580 (m). For NMR data and analysis, see [33].

#### 3.7.7. *N*-(3-Hydroxyphenyl)-1,4-dideoxy-1,4-imino-l-gulitol **meta 7** (Scheme 1, step (vi))

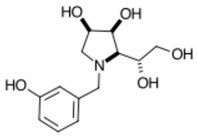



*N*-(3-Methylphenyl boronic acid)-3,6-dideoxy-3,6-imino-d-glucofuranose **meta 3** (61 mg, 0.205 mmol) was dissolved in EtOH (aq 50%, 10 mL) prior to the addition of NaBH_4_ (9 mg, 0.246 mmol, 1.2 eq) and the reaction was allowed to stir at room temperature for 30 minutes. Additional NaBH_4_ (11 mg, 0.298 mmol, 1.5 eq) was added and the reaction stirred for an additional 1 hour. the reaction was observed to be incomplete so additional NaBH_4_ (9 mg, 0.246 mmol, 1.2 eq) was added and the reaction stirred at room temperature for 18 hours, after which the reaction was quenched with glacial acetic acid (a few drops) and dried to give a crude mass (112 mg). A portion (52 mg) was purified by prep t.l.c. (toluene/acetone/acetic acid, 10:15:1) which resulted in oxidation to give **meta 7** (39 mg, 0.144 mmol, quant.) as a yellow residue. [α]_289_^19^ 24.65 (c 0.052 in H_2_O); ν_max_ (thin film, cm^-1^): 3377 (s, broad, OH), 2964 (w, alkyl CH), 1560 (s, 1489 (w), 1413 (s, ArC=C), 1347 (w), 1285 (w, C-O), 1206 (w), 1179 (m, OH bend), 1125 (s), 1038 (m), 1014 (w), 963 (w), 816 (w), 755 (w, meta-subst), 687 (w), 653 (w, meta-subst), 620 (w), 568 (w), 471 (w), 416 (w); δ_H_ (Acetic acid-*d*^4^, 400MHz): 7.39 (1H, ddd, partially obscured, *J*_HE,HD_ 7.2 Hz, *J*_HE,HC_ 2.5, *J*_HE,HA_ 0.8 Hz, ArH^E^), 7.09 (1H, dt, *J*_HD,HC_ 7.8, *J*_HD,HA_ 1.0 Hz, ArH^D^), 7.02 (1H, app-d, *J* 1.1 Hz, ArH^A^), 7.01 (1H, ddd, partially obscured, *J*_HC,HD_ 7.5 Hz, *J*_HC,HE_ 2.5 Hz, *J*_HC,HA_ 0.8 Hz, ArH^D^), 4.66 (1H, d, *J*_Ha,Hb_ 13.0 Hz, ArC*H^a^*H^b^), 4.49 (1H, ddd, *J*_H-2,H-1_ 8.7, *J*_H-2,H-1′_ 7.0, *J*_H-2,H-3_ 4.0 Hz, H-2), 4.39 (1H, app-t, *J*_H-3,H-2/4_ 4.3 Hz, H-3), 4.32 (1H, ddd, *J*_H-5,H-4_ 8.8, *J*_H-5,H-6_ 5.2, *J*_H-5,H-6′_ 3.3 Hz, H-5), 4.17 (1H, d, *J*_Hb,Ha_ 13.0 Hz, ArCH^a^*H^b^*), 3.82 (1H, dd, *J*_H-6′,H-6_ 12.2, *J*_H-6′,H-5_ 3.2 Hz, H-6′), 3.74 (1H, dd, *J*_H-4,H-5_ 9.1, *J*_H-4,H-3_ 4.7 Hz, H-4), 3.68 (1H, dd, *J*_H-6,H-6′_ 12.1, *J*_H-6,H-5_ 5.2 Hz, H-6), 3.48 (1H, dd, *J*_H-1′,H-1_ 12.0, *J*_H-1,H-2_ 7.0 Hz, H-1′), 3.23 (1H, dd, partially obscured, *J*_H-1,H-1′_ 12.0, *J*_H-1,H-2_ 8.4 Hz, H-1); δ_C_ (Acetic acid-*d^4^*, 100MHz): 152.8 (ArC_quat_-OH), 127.4 (C^E^), 126.7 (ArC_quat_), 119.5 (C^D^), 114.3 (C^A^), 113.5 (C^C^), 67.1 (C-3), 66.3 (C-4), 65.8 (C-2), 65.5 (C-5), 59.8 (C-6), 57.4-58.2 (ArCH_2_, 81 Hz), 50.3 (C-1). δ_B_ (Acetic acid-*d^4^*, 128 MHz): No signal.

#### 3.7.8. *N*-(3-Methylphenyl boronic acid)-3,6-dideoxy-3,6-imino-1,2-*O*-isopropylidene-α-d-glucofuranose **meta 5** (Scheme 1, step (iv)) and *N*-(3-hydroxyphenyl)-3,6-dideoxy-3,6-imino-1,2-*O*-isopropylidene-α-d-gulofuranose **meta 6** (Scheme 1, step (v)) 

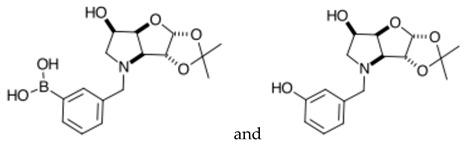



3,6-Dideoxy-3,6-imino-1,2-*O*-isopropylidene-α-d-glucofuranose-*p*-toluenesulfonate **1** (98 mg, 0.268 mmol, 1.1 eq) was dissolved in DMF (10 mL) prior to the addition of K_2_CO_3_ (34 mg, 0.243 mmol, 1.0 eq) and 3-bromomethylphenyl boronic acid (52 mg, 0.243 mmol, 1.0 eq). The reaction was heated to 100 °C and allowed to stir for 19 hours. T.l.c. analysis (EtOAc) showed presence of one product (R*_f_* 0.68) and consumption of the starting material (R*_f_* 0.00). The solvent was evaporated and the crude dissolved in CHCl_3_ (60 mL) and washed with NaOH (aq. 0.1 M, 20 mL) to give *N*-(3-methylphenyl boronic acid)-3,6-dideoxy-3,6-imino-1,2-*O*-isopropylidene-α-d-glucofuranose **meta 5** in the organic layer as a brown oil (88 mg, 0.263 mmol, 98%). [α]_289_^19^ 0.13 (c 0.072 in MeOH). ν_max_ (thin film, cm^-1^): 3441, 3415, 3398, 3309, 3283 (s, broad), 1681, 1656 (m), 1601, 1508, 1473, 1458, *1450*, 1436 (w, ArC=C), 1385 (w, B-O), 1185 (s, OH bend), 1126 (s), 1040 (s), 1012 (s), 814, 719, 710, 683 (m), 617 (m), 566 (s), 514, 495, 454, 424 (m). (Scheme 1, step (v)). When **meta 5** is left on the bench or aqueous solution, it slowly oxidises to *N*-(3-hydroxyphenyl)-3,6-dideoxy-3,6-imino-1,2-*O*-isopropylidene-α-d-gulofuranose **meta 6**. δ_H_ (Acetic acid-*d^4^*, 400 MHz): 8.09 (1H, s, ArH^A^), 7.94 (1H, d, *J*_HC,HD_ 7.5 Hz, ArH^C^), 7.76 (1H, d, *J*_HE,HD_ 7.8 Hz, ArH^E^), 7.48 (1H, app-t, *J*_HD,HE/HC_ 7.6 Hz, ArH^D^), 6.15 (1H, d, *J* 2.5 Hz, H-1), 5.09-4.96 (2H, app-s, H-2 and H-4), 4.68-4.58 (1H, obscured, H-5), 4.62 (1H, d, *J*_Ha,Hb_ 11.8 Hz, ArC*H^a^*H^b^), 4.55 (1H, d, *J*_Hb,Ha_ 11.8 Hz, ArCH^a^*H^b^*), 4.28 (1H, d, *J* 4.3 Hz, H-3), 3.74 (1H, dd, *J*_H-6,H-6′_ 12.6 Hz, *J*_H-6,H-5_ 5.2 Hz, H-6), 3.54 (1H, dd, *J*_H-6′,H-6_ 12.2 Hz, *J*_H-6′,H-5_ 5.1 Hz, H-6′), 1.45, 1.27 (6Hs, 2 × s, 2 × acetonide CH_3_); δ_C_ (Acetic acid-*d^4^*, 100 MHz): 148.5 (ArC_quat_-OH), 138.1 (ArC^A^), 136.8 (ArC^C^), 134.6 (ArC^E^), 129.6 (ArC^D^), 114.4 (acetonide C_quat_), 108.5 (C-1), 82.9 (C-4 and C-2), 74.0 (C-3), 69.7 (C-5), 60.0 (ArCH_2_), 59.2 (C-6), 27.3, 26.8 (2 × CH_3_ acetonide). ArC_quat_ not discernible. δ_B_ (Acetic acid-*d^4^*, 128 MHz): No signal.

## 4. Conclusions

BNCT is the least destructive radiation therapy to the human body, provided BNCT agents can be selectively delivered to cancer cells. Current clinical agents based on inorganic boron moieties do not yet possess an ideal profile in terms of toxicity, membrane-crossing capabilities and accumulation into cancer cells. We provide the first examples of monosaccharides (and their congeners) borylated (new B-C bond) with organic boron moieties to improve toxicity and accumulation profiles, with a view to improved ADME profiles and a more targeted/controllable radiation delivery. This paper communicates another synthetic development and biological investigation to borylated pyrrolidine iminosugars, and continues to cement the opening of the field of borylated carbohydrates for controllable switch on/switch off cancer radiotherapies. It is envisaged this type of cancer therapy technology could in the future be incorporated in long-term manned space travels.

This paper provides a complete set of structure–activity relationship data for this novel family of boron-bearing iminosugars displaying selective, moderate-to-weak inhibitions (IC_50_s = 116–617 μM) of β-d-galactosidase (bovine liver), and emerging inhibition of β-d-glucosidases (almond, bovine liver) (IC_50_s = 633 and 710 μM) and α-d-glucosidases (rice, yeast, rat intestinal maltase) (IC_50_s = 106–784 μM). This indicates the boronic acid pharmacophore may form suitable interactions for management of lysosomal storage disorders to support restoration of biological activity of mutant enzymes via the chaperone-mediated therapy approach. From a structure–activity perspective, the cancer screening revealed that the A2780 ovarian carcinoma cell line is selectively inhibited by all compounds screened and the MIA-Pa-Ca2 pancreatic carcinoma cell line is also selectively inhibited. The real therapeutic potential of these borylated drugs lies in their controllable switch on/switch off activation under BNCT radiotherapeutic conditions.

## Figures and Tables

**Table 1:** Summary of glycosidase inhibitions by *N*-benzyl-1,4-dideoxy-1,4-imino derivatives.

Iminosugar	Most Inhibited Glycosidase/s
*N*-Benzyl-1,4-dideoxy-1,4-imino-	
d-allitol [43]	α-l-Fucosidase (76%)
d-galactitol [44]	α-d-Glucosidase (93.2%), IC_50_ = 40.6 μM
l-gulitol **5** [30]	NI
d-mannitol.HCl [45,46,47]	α-d-Mannosidases (lysosomal acidic, 34%), (neutral, 44%) and (Golgi II, 72%)
d-talitol.HCl [48]	NI
*N*-(2-Methylphenyl boronic acid)-1,4-dideoxy-1,4-imino-d-talitol.HCl [48]	β-d-Galactosidase (*E. coli*) (44–55%)
*N*-Benzyl-3,6-dideoxy-3,6-imino-d-gulofuranose **4** [30]	β-d-Galactosidase (bovine liver), IC_50_ = 133 μM
*N*-(4-Methylphenyl boronic acid)-3,6-dideoxy-3,6-imino-d-gulofuranose **para 7** [30]	β-d-Galactosidase (bovine liver), IC_50_ = 218 μM
*N*-(4-Methylphenyl boronic acid)-1,4-dideoxy-1,4-imino-l-gulitol **para 8** [30]	β-d-Galactosidase (bovine liver), IC_50_ = 501 μM

## Data Availability

Data presented in this study is contained within the article and Supplementary Material. Further inquiries can be directed to the corresponding author.

## Supplementary Materials

The following supporting information can be downloaded at https://www.mdpi.com/article/10.3390/ph18111739/s1, Figure S1: ^1^H- (400 MHz), ^13^C-NMR (100 MHz), DEPT, COSY and HSQC spectra of *N*-(3-hydroxyphenyl)-3,6-dideoxy-3,6-imino-1,2-*O*-isopropylidene-α-d-gulofuranose **meta 6** in *Acetic acid-d^6^*.; Figure S2: ^1^H- (400 MHz), ^13^C-NMR (100 MHz), DEPT, COSY, HSQC and HMBC spectra of *N*-(3-hydroxyphenyl)-1,4-dideoxy-1,4-imino-l-gulitol **meta 7** in *Acetic acid-d^6^*.
