# Supplementary material for: Borylated 5-Membered Ring Iminosugars: Synthesis and Biological Evaluation for Glycosidase Inhibition and Anticancer Properties for Application in Boron Neutron Capture Therapy (BNCT)—Part 2
Source: Pharmaceuticals (Basel). 2025 Nov 17;18(11):1739. doi: 10.3390/ph18111739 (PMC12655172; doi:10.3390/ph18111739)
Supplement: Supplementary file 1 [file pharmaceuticals-18-01739-s001.zip › pharmaceuticals-3880625-supplementary.pdf]

Supplementary Information

Borylated 5-membered Ring Iminosugars: Synthesis and Biological Evaluation for Glycosidase Inhibition and Anticancer Properties for Application in Boron Neutron Capture Therapy (BNCT) – Part 2

Kate Prichard [1], Kosuke Yoshimura [2], Suzuka Yamamoto [2], Atsumi Taguchi [2], Barbara Bartholomew [3], Jayne Gilbert [4], Jennette Sakoff [4], Robert Nash [3], Atsushi Kato [2], Michela Simone [1,*,†]

Index

Figure S1. ^{1}H- (400 MHz), ^{13}C-NMR (100 MHz), DEPT, COSY and HSQC spectra of *N*-(3-hydroxyphenyl)-3,6-dideoxy-3,6-imino-1,2-*O*-isopropylidene-α-D-gulofuranose **meta 6** in *Acetic acid-d*^{6}. 2

Figure S2. ^{1}H- (400 MHz), ^{13}C-NMR (100 MHz), DEPT, COSY, HSQC and HMBC spectra of *N*-(3-hydroxyphenyl)-1,4-dideoxy-1,4-imino-L-gulitol **meta 7** in *Acetic acid-d*^{6}. 12

NMR Experimental details (*Acetic acid-d*^{6}). 25

Figure S1. ^{1}H- (400 MHz), ^{13}C-NMR (100 MHz), DEPT, COSY and HSQC spectra of *N*-(3-hydroxyphenyl)-3,6-dideoxy-3,6-imino-1,2-*O*-isopropylidene-*α*-D-gulofuranose **meta 6** in *Acetic acid-d*[6].

^{1}H-NMR

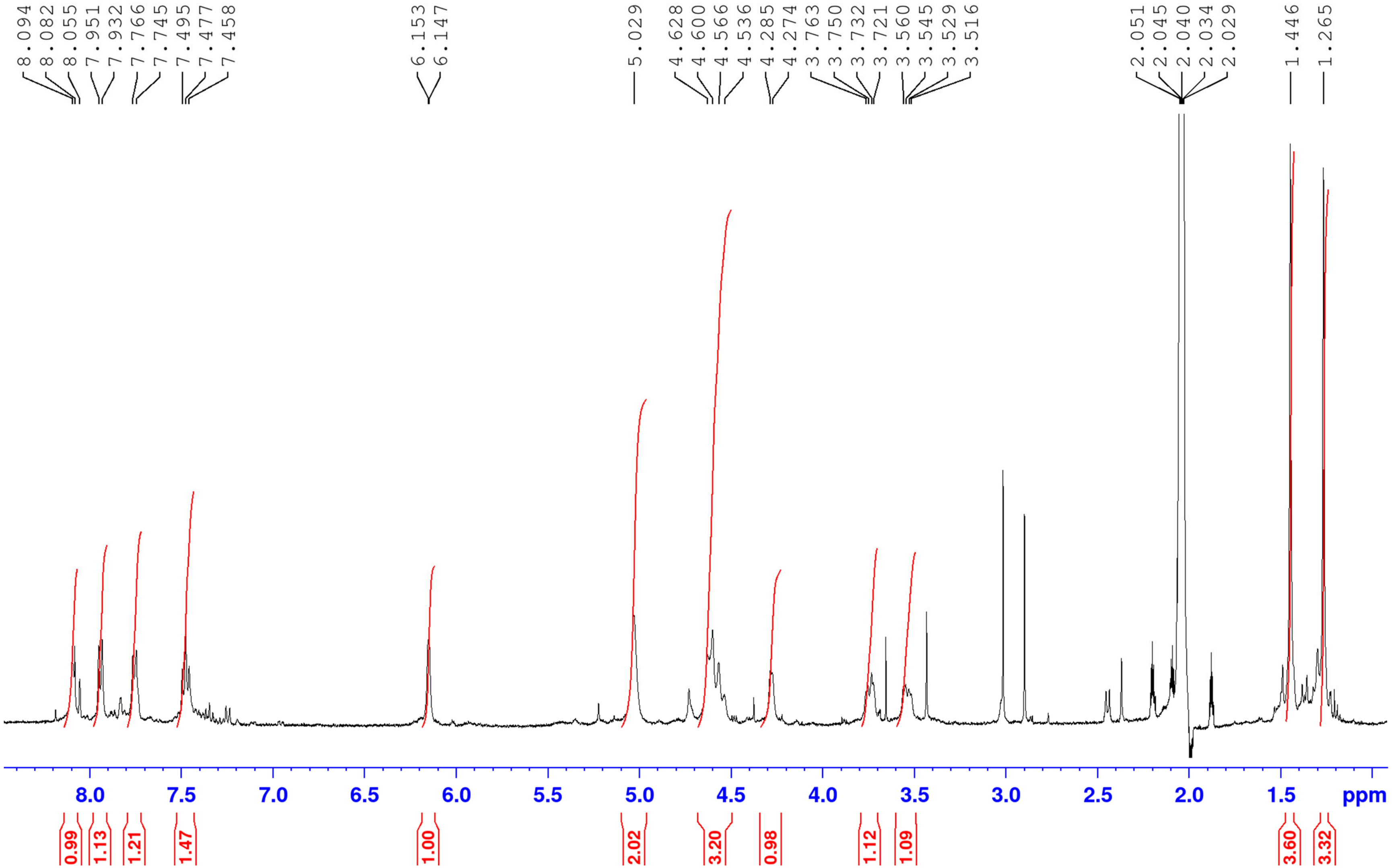

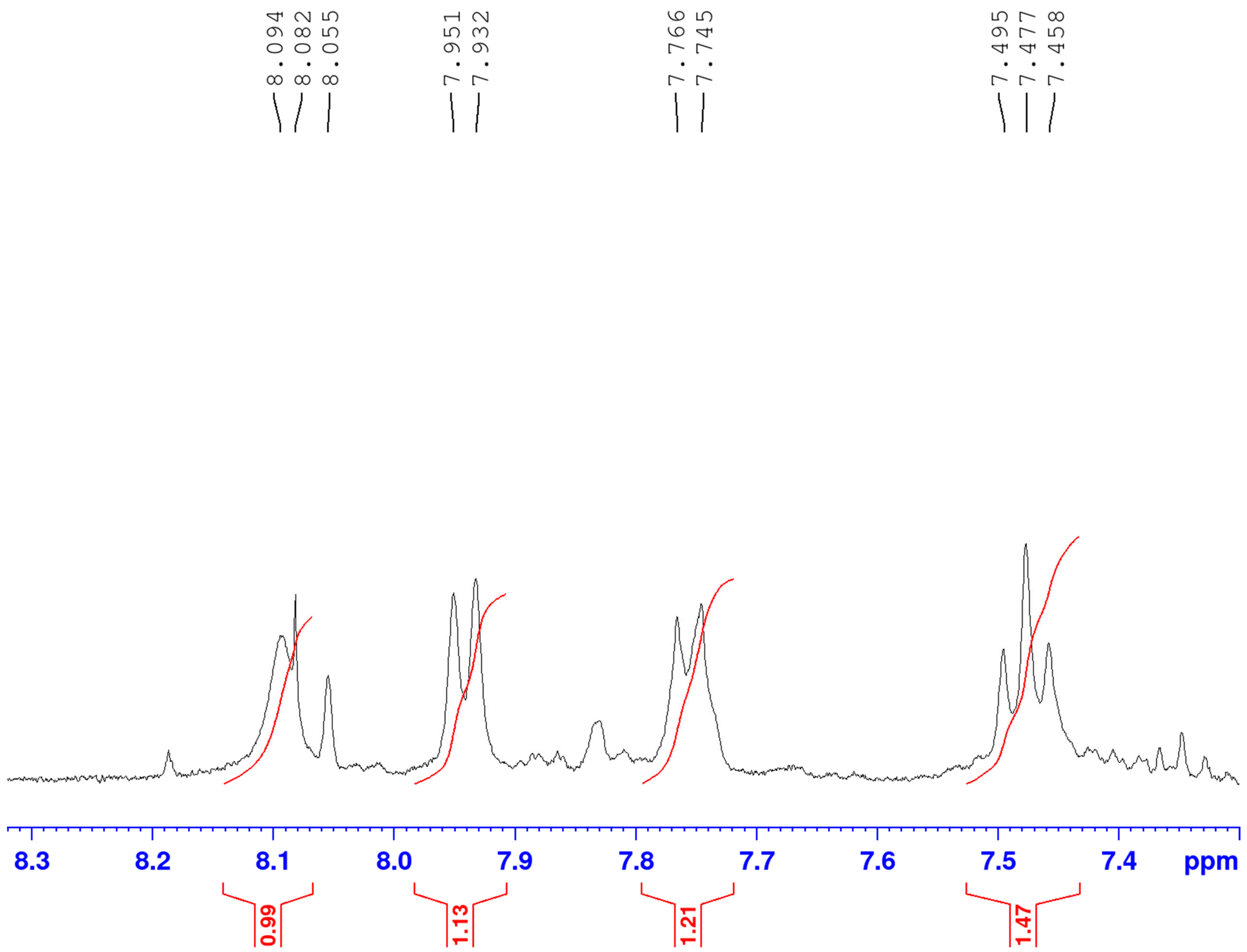
8.094
8.082
8.055
7.951
7.932
7.766
7.745
7.495
7.477
7.458
8.3
8.2
8.1
8.0
7.9
7.8
7.7
7.6
7.5
7.4
ppm
0.99
1.13
1.21
1.47

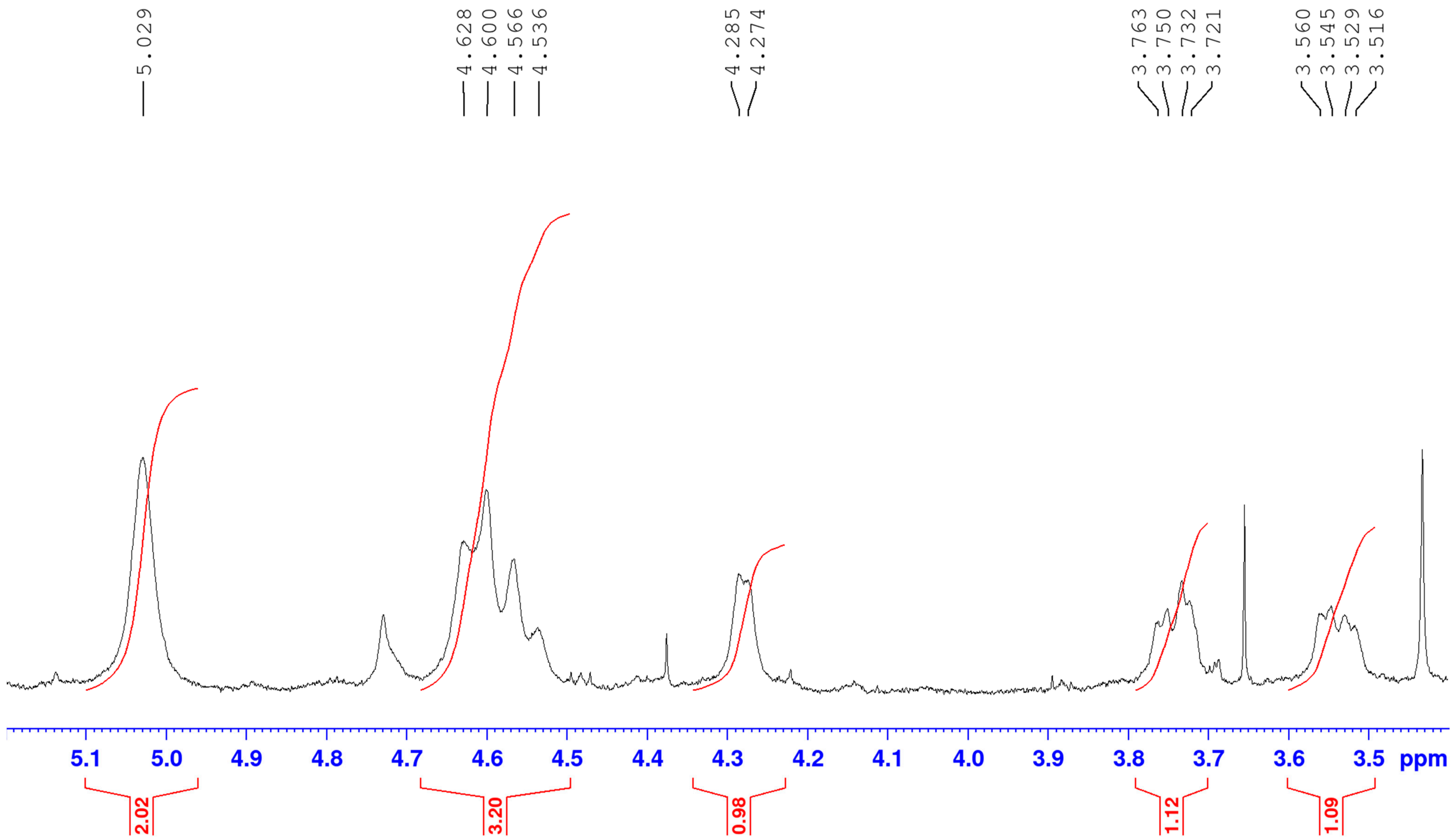
5.029
4.628
4.600
4.566
4.536
4.285
4.274
3.763
3.750
3.732
3.721
3.560
3.545
3.529
3.516
5.1
5.0
4.9
4.8
4.7
4.6
4.5
4.4
4.3
4.2
4.1
4.0
3.9
3.8
3.7
3.6
3.5
ppm
2.02
3.20
0.98
1.12
1.09

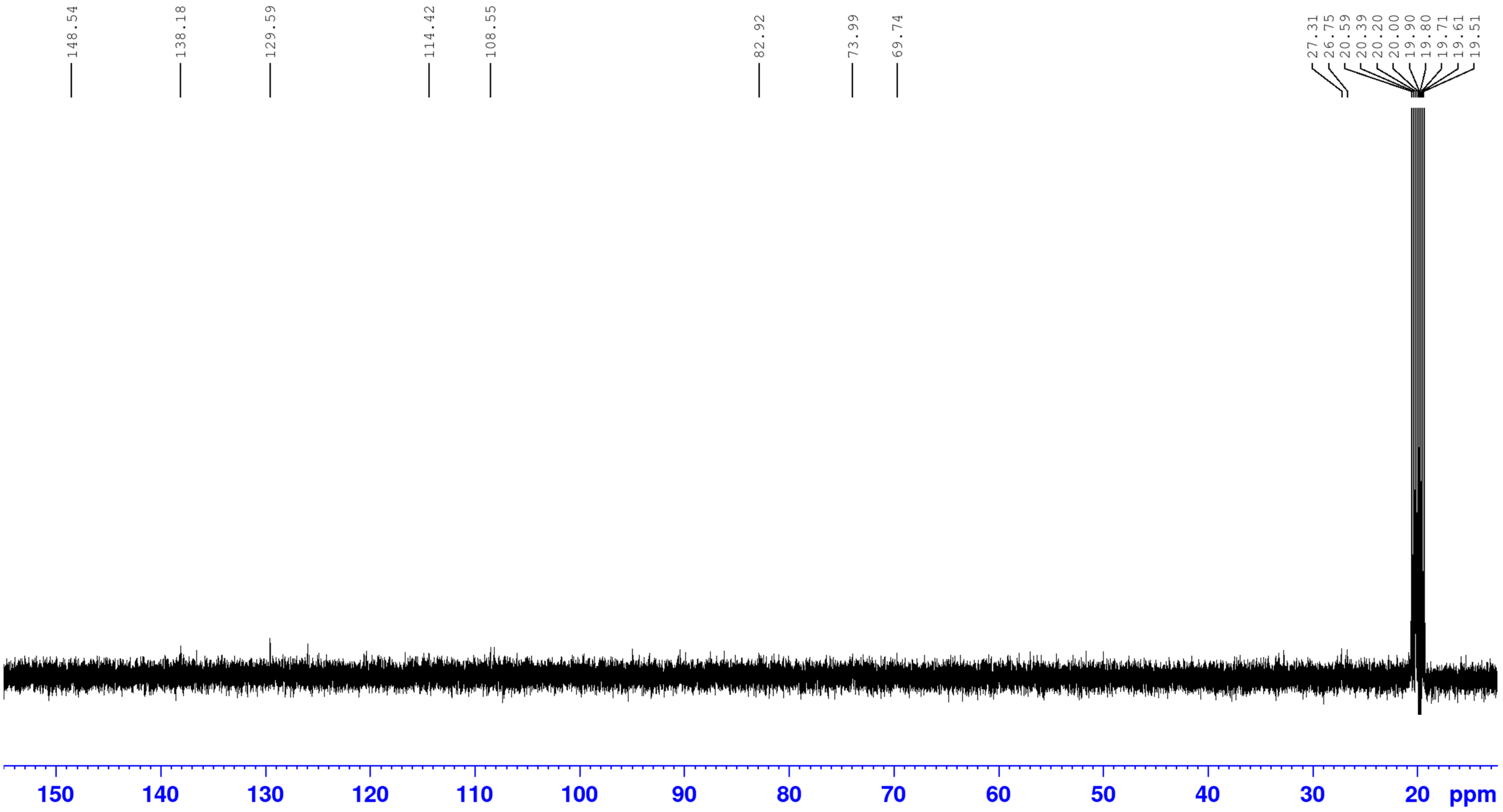
148.54
138.18
129.59
114.42
108.55
82.92
73.99
69.74
27.31
26.75
20.59
20.39
20.20
20.00
19.90
19.80
19.71
19.61
19.51
150
140
130
120
110
100
90
80
70
60
50
40
30
20
ppm

^{11}B-NMR

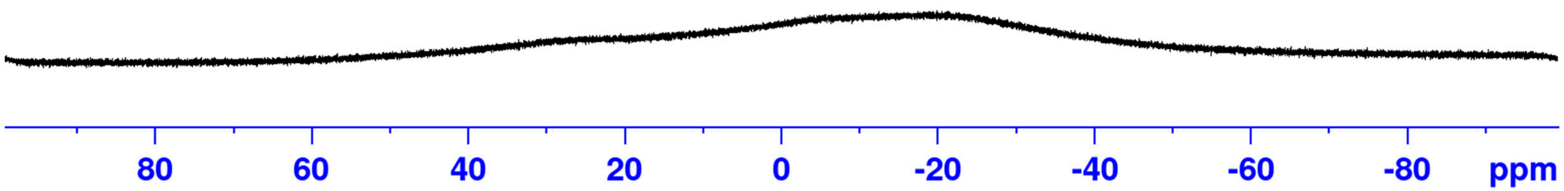

COSY

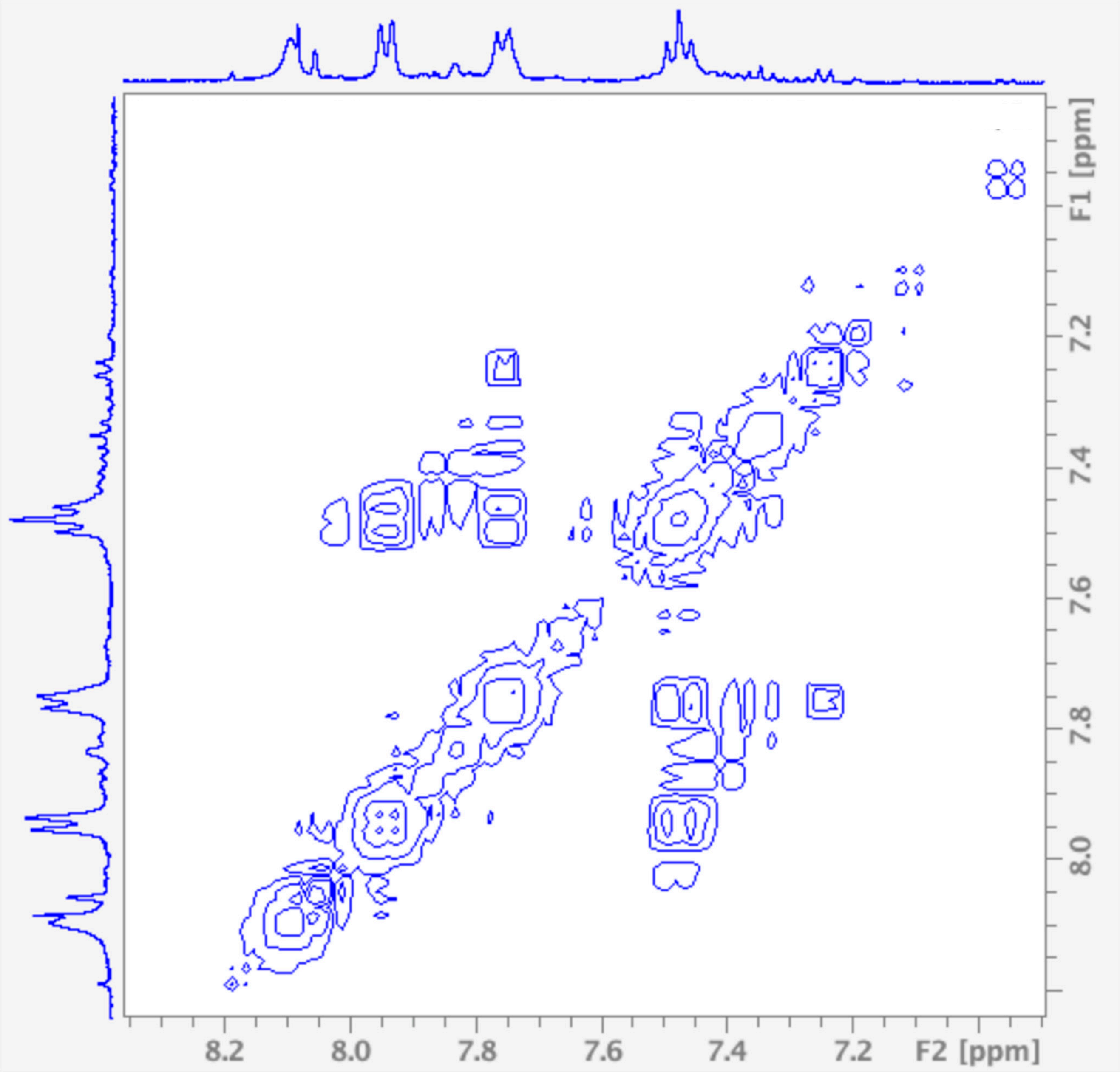

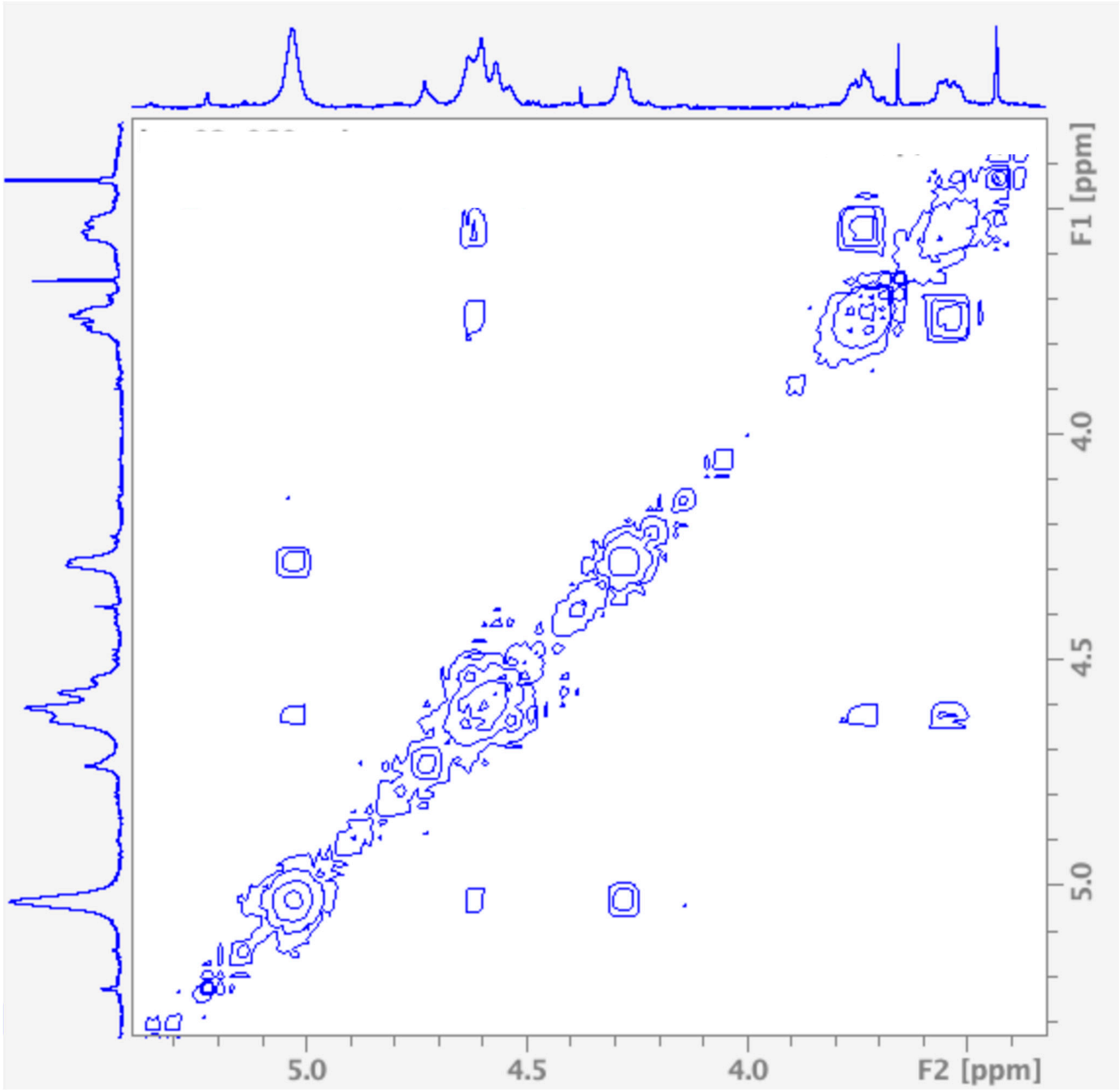

F1 [ppm]
4.0
4.5
5.0
F2 [ppm]
5.0
4.5
4.0

HSQC

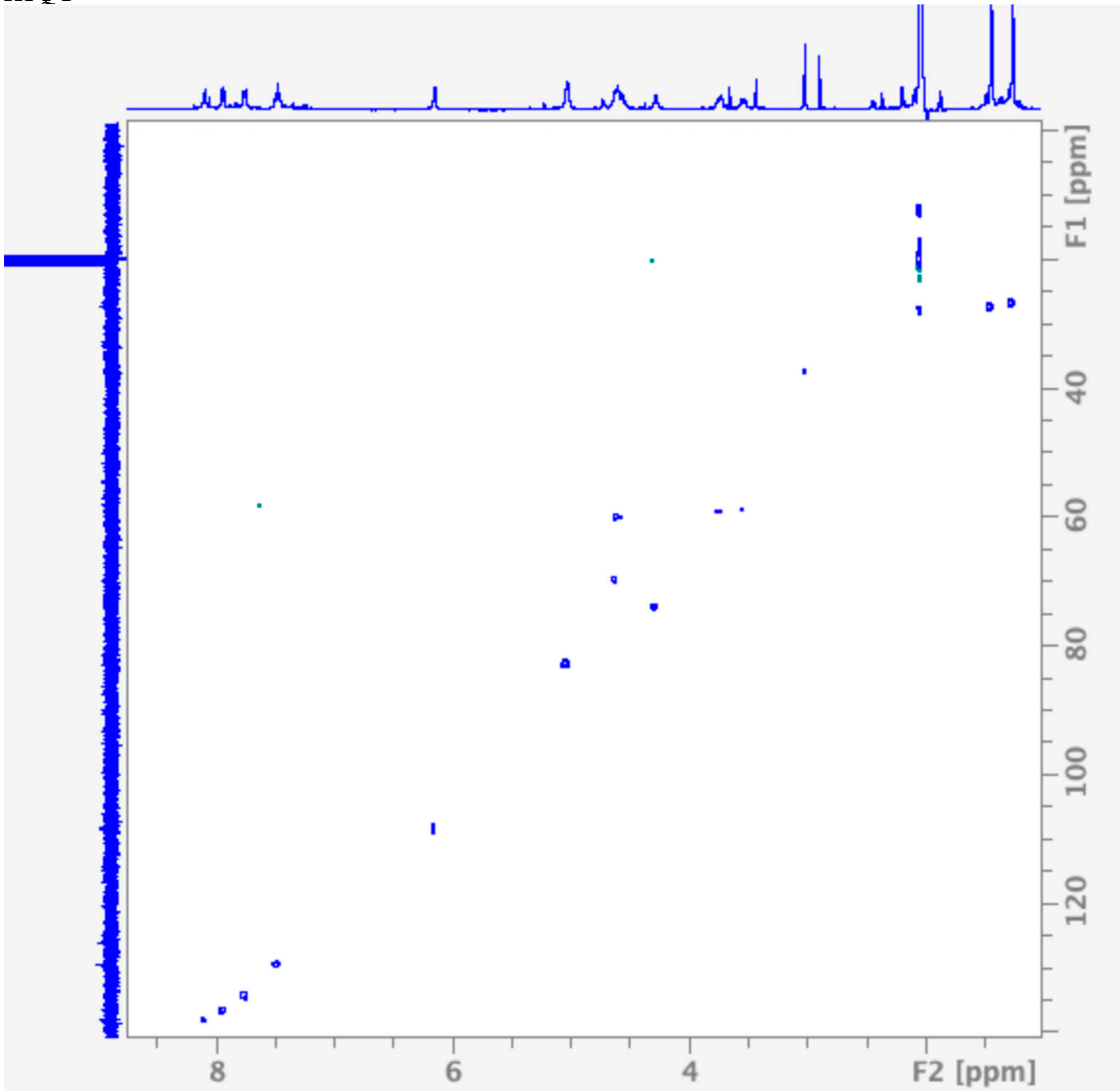

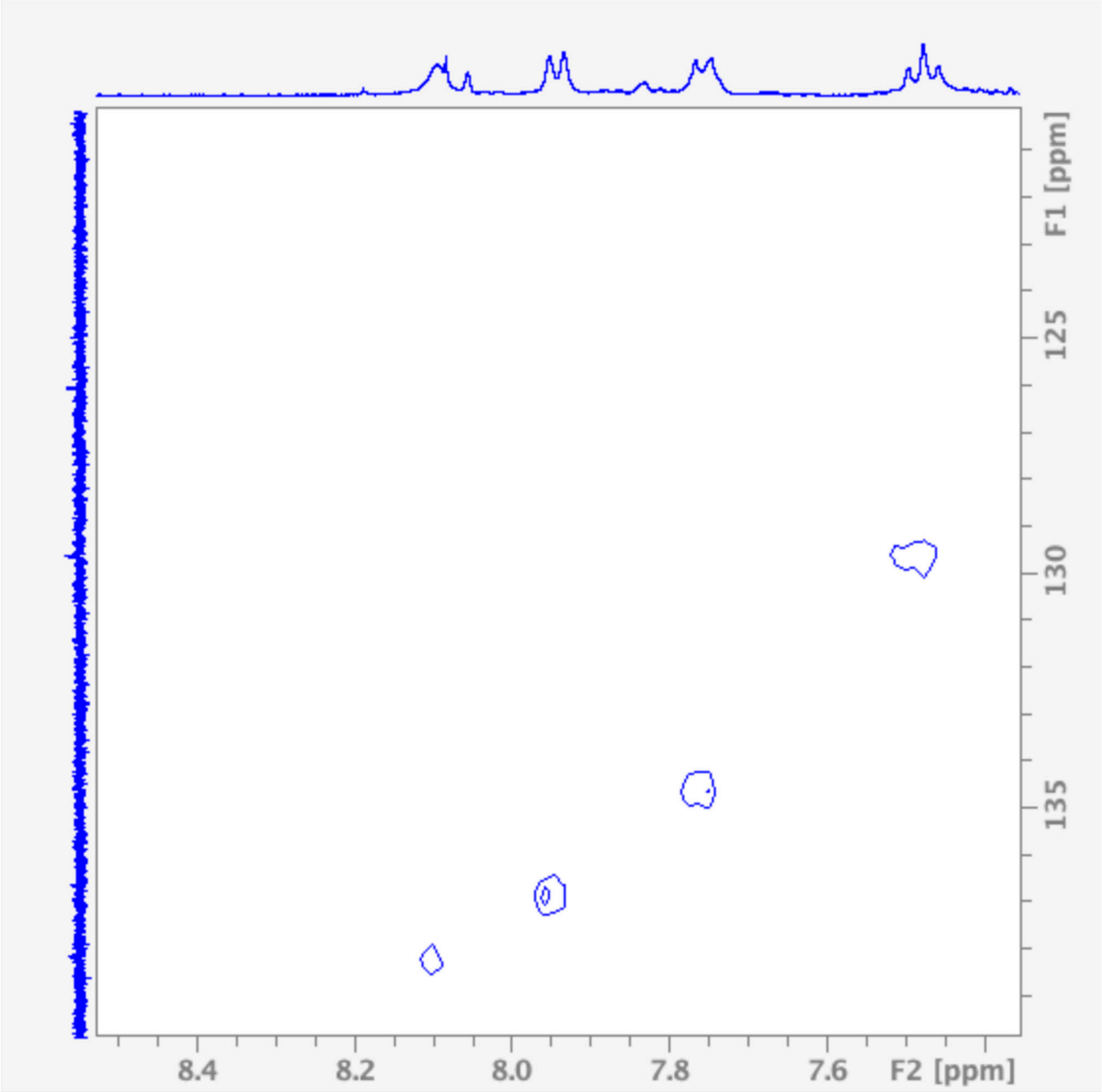
F1 [ppm]
125
130
135
8.4
8.2
8.0
7.8
7.6
F2 [ppm]

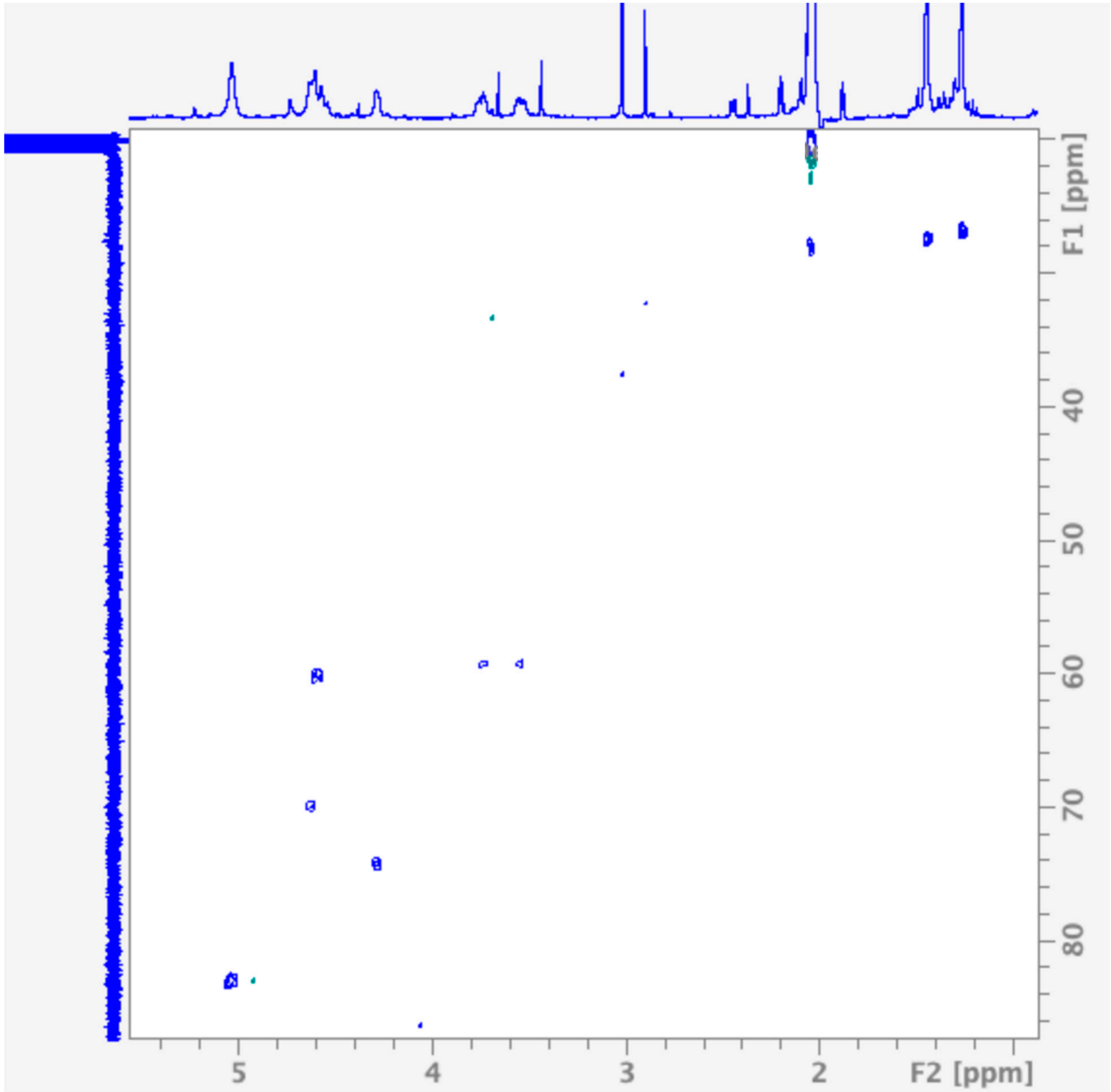

F1 [ppm]
40
50
60
70
80
5
4
3
2
F2 [ppm]

Figure S2. ^{1}H- (400 MHz), ^{13}C-NMR (100 MHz), DEPT, ^{11}B-NMR (128 MHz), COSY and HSQC spectra of *N*-(3-hydroxyphenyl)-1,4-dideoxy-1,4-imino-L-gulitol **meta 7** in *Acetic acid-d*^{6}.

^{1}H-NMR

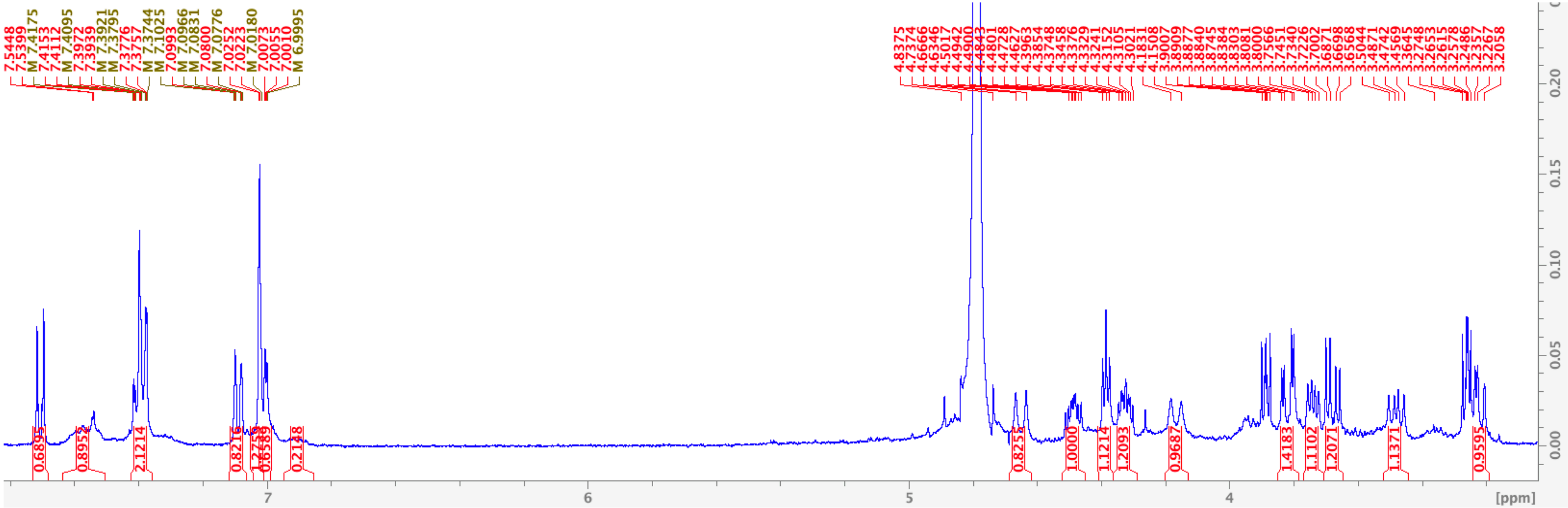

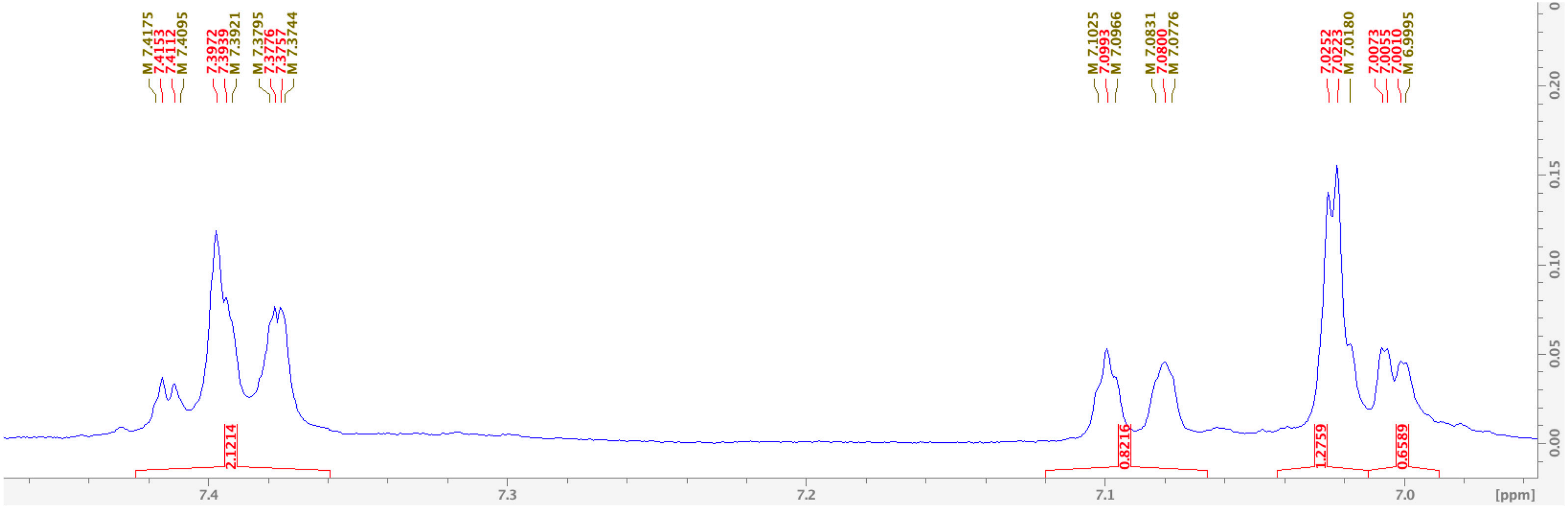
M 7.4175
7.4153
7.4112
M 7.4095
7.3972
7.3939
M 7.3921
M 7.3795
7.3776
7.3757
M 7.3744
M 7.1025
7.0993
M 7.0966
M 7.0831
7.0800
M 7.0776
7.0252
7.0223
M 7.0180
7.0073
7.0055
7.0010
M 6.9995
2.1214
0.8216
1.2759
0.6589
7.4
7.3
7.2
7.1
7.0
[ppm]
0
0.20
0.15
0.10
0.05
0.00

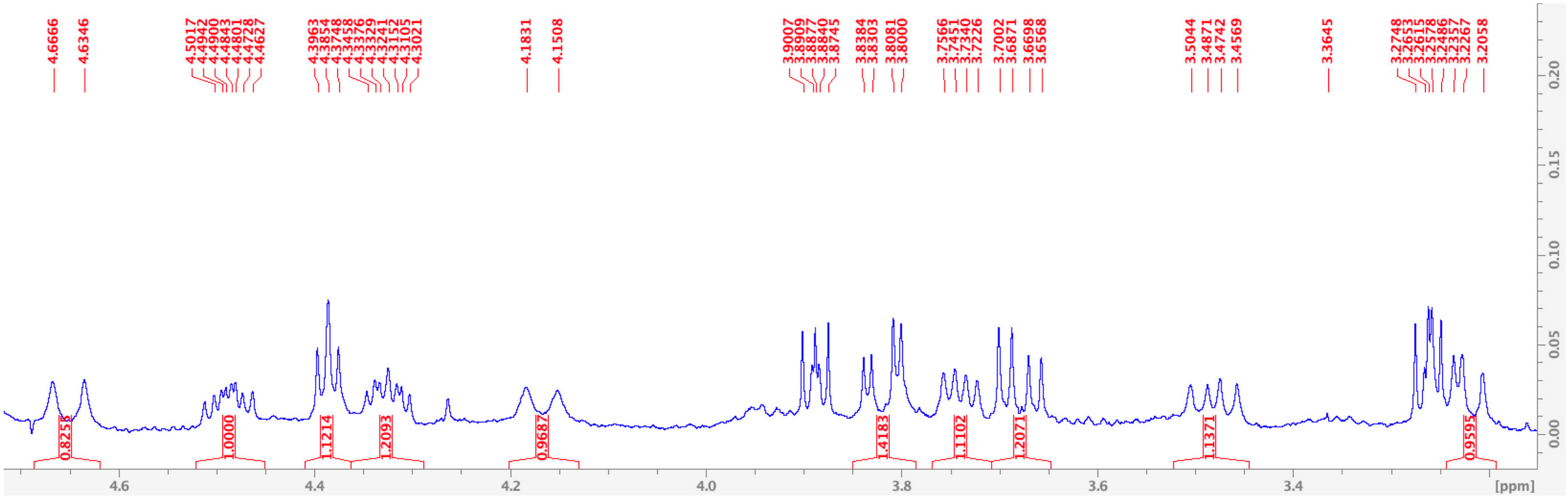

^{13}C-NMR

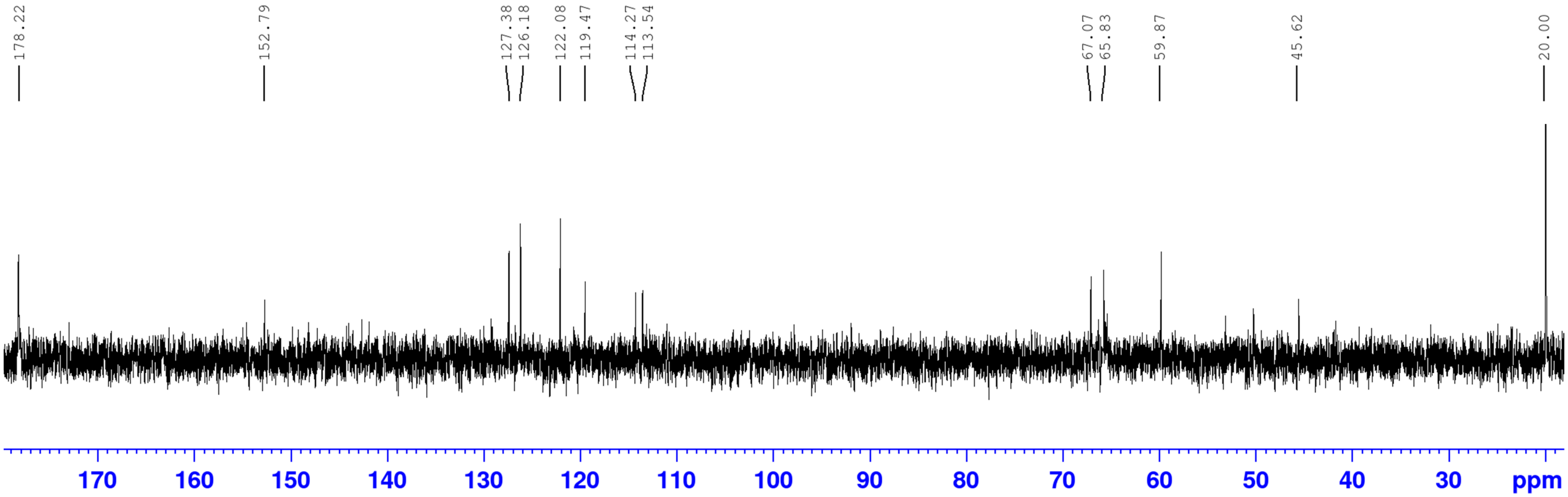

178.22
152.79
127.38
126.18
122.08
119.47
114.27
113.54
67.07
65.83
59.87
45.62
20.00
170
160
150
140
130
120
110
100
90
80
70
60
50
40
30
ppm

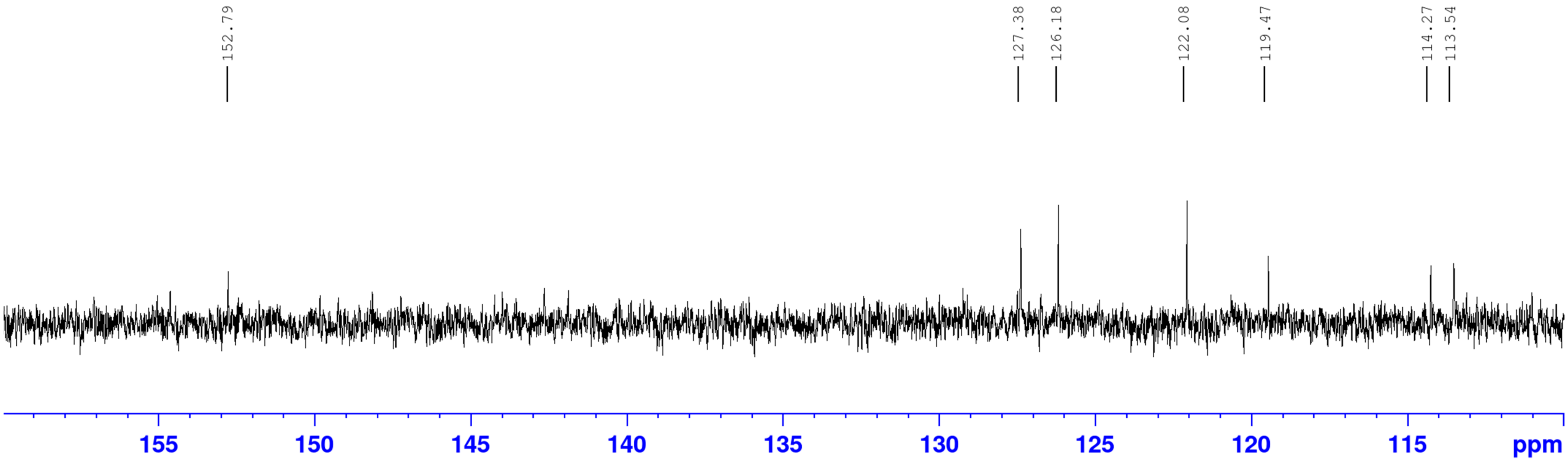
152.79
127.38
126.18
122.08
119.47
114.27
113.54
155
150
145
140
135
130
125
120
115
ppm

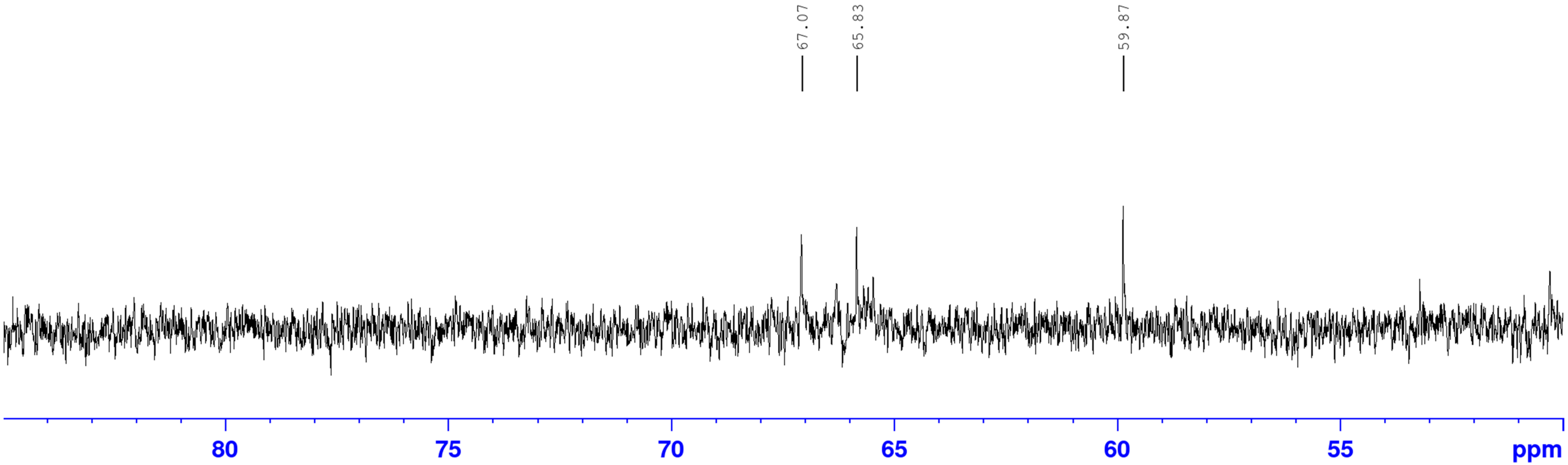
67.07
65.83
59.87
80
75
70
65
60
55
ppm

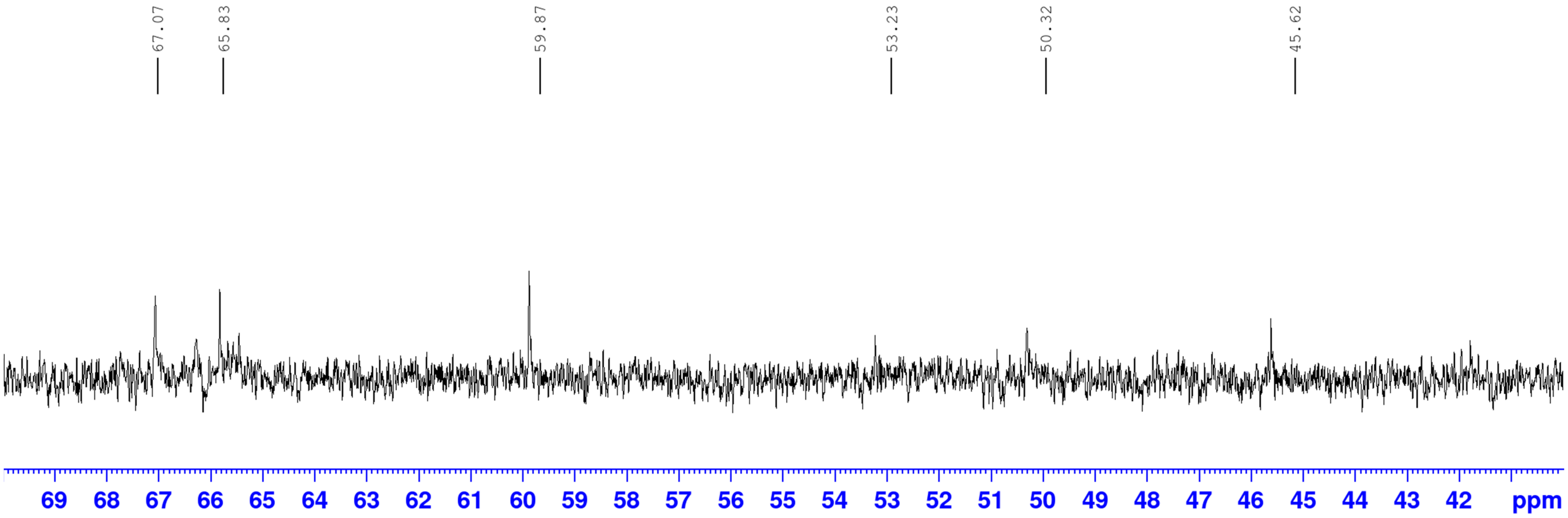

67.07
65.83
59.87
53.23
50.32
45.62
69
68
67
66
65
64
63
62
61
60
59
58
57
56
55
54
53
52
51
50
49
48
47
46
45
44
43
42
ppm

`

DEPT

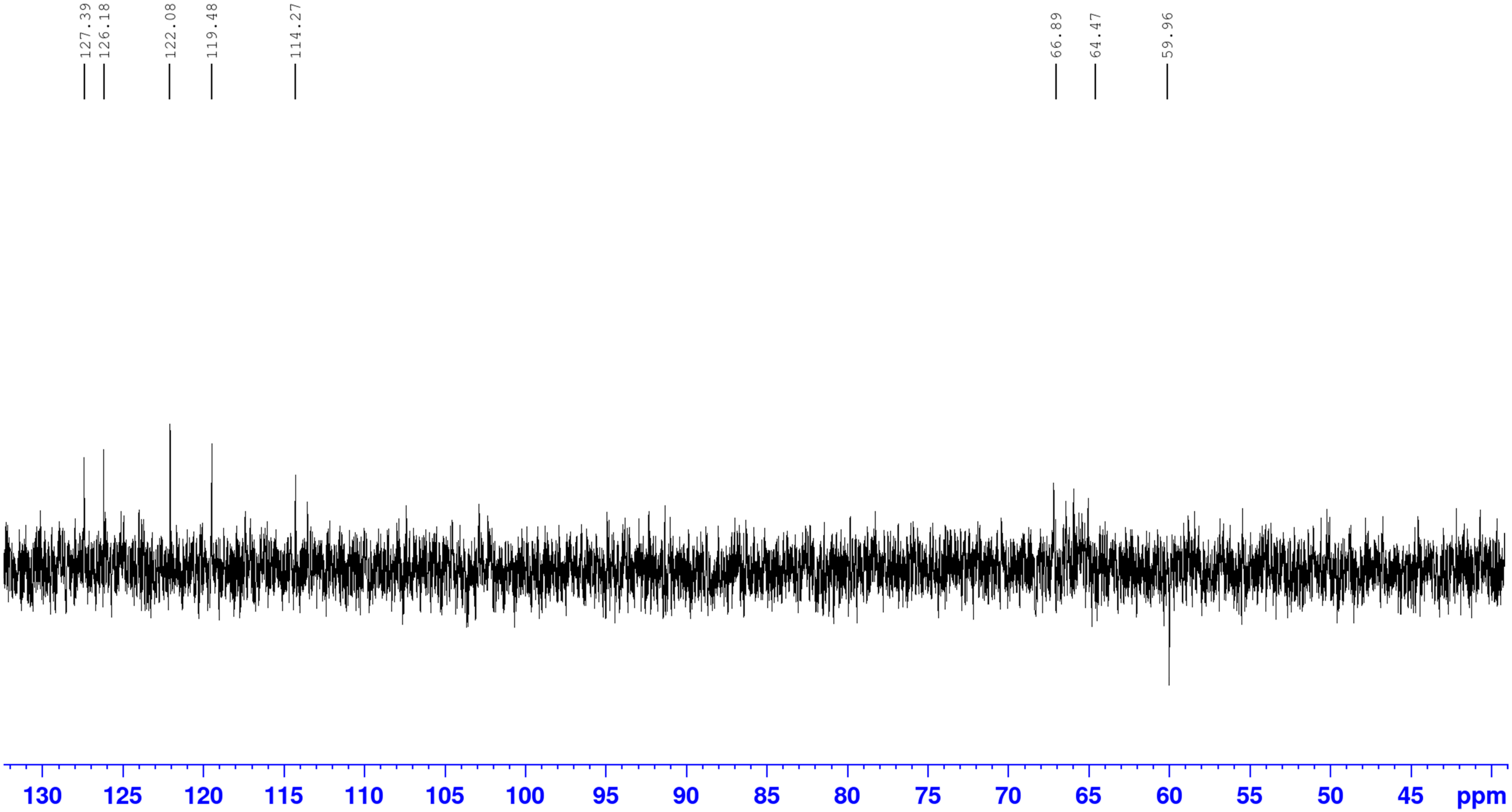
127.39
126.18
122.08
119.48
114.27
66.89
64.47
59.96
130
125
120
115
110
105
100
95
90
85
80
75
70
65
60
55
50
45
ppm

^{11}B-NMR

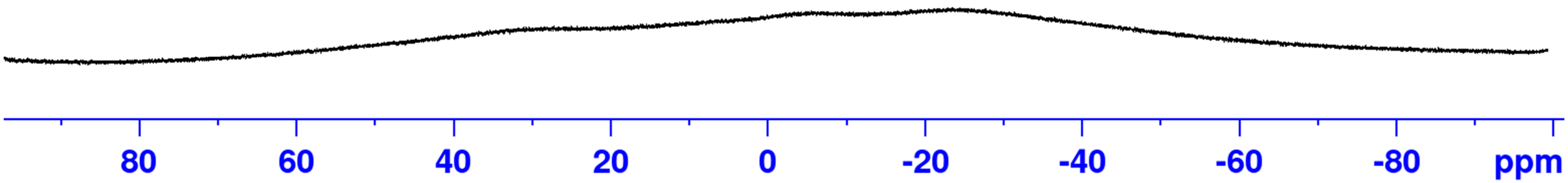

COSY

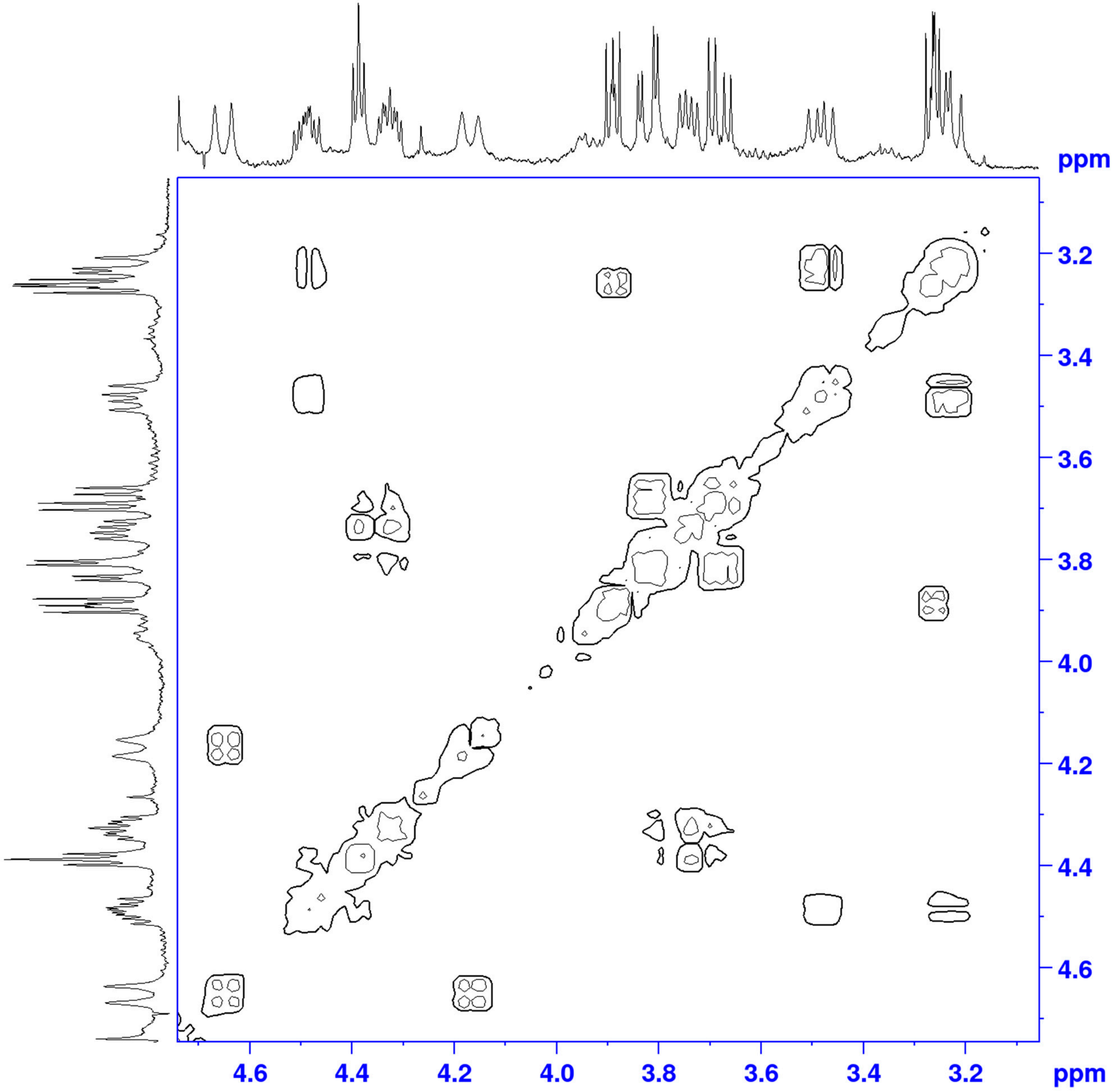

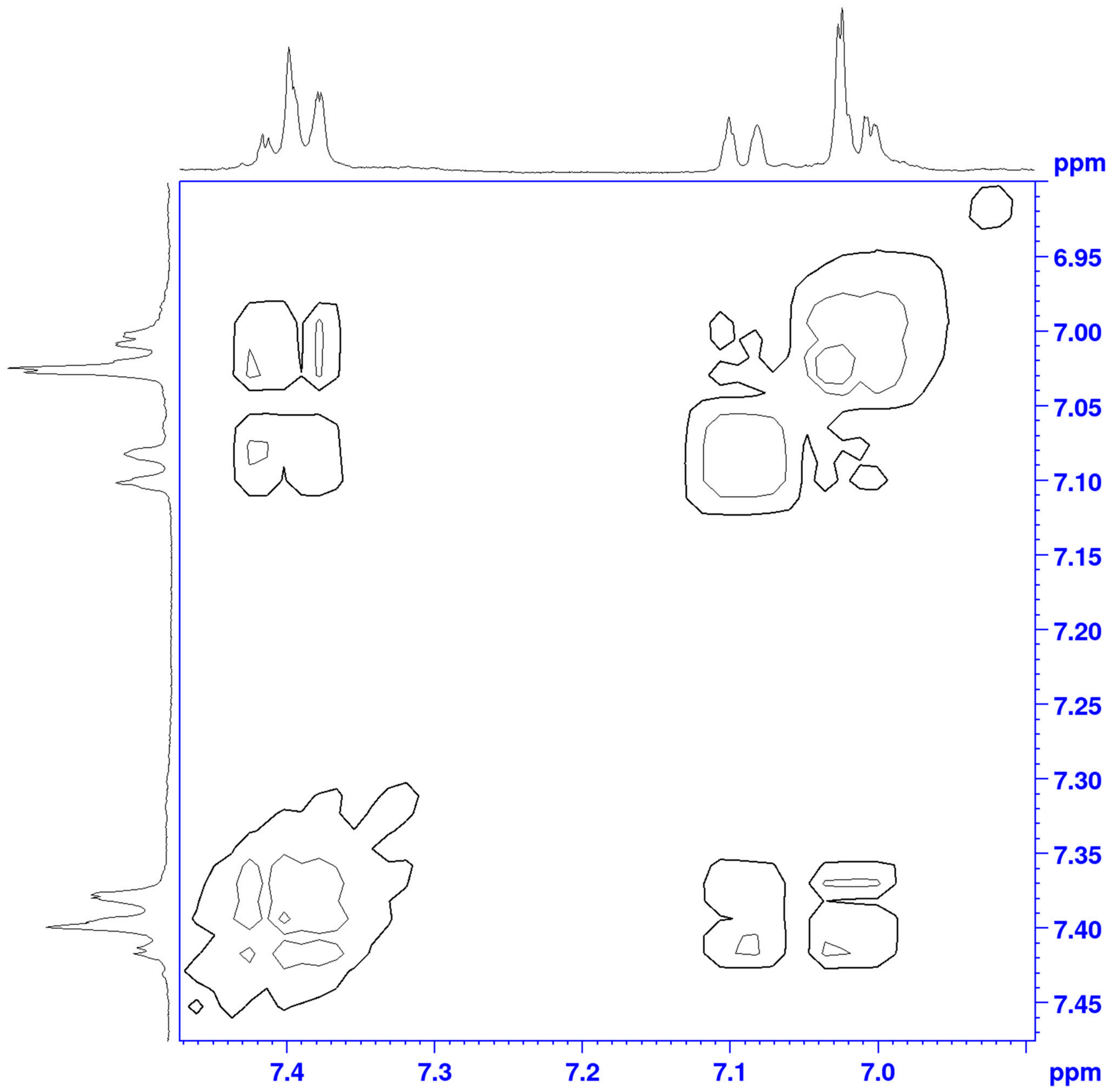

ppm
6.95
7.00
7.05
7.10
7.15
7.20
7.25
7.30
7.35
7.40
7.45
7.4
7.3
7.2
7.1
7.0
ppm

HSQC

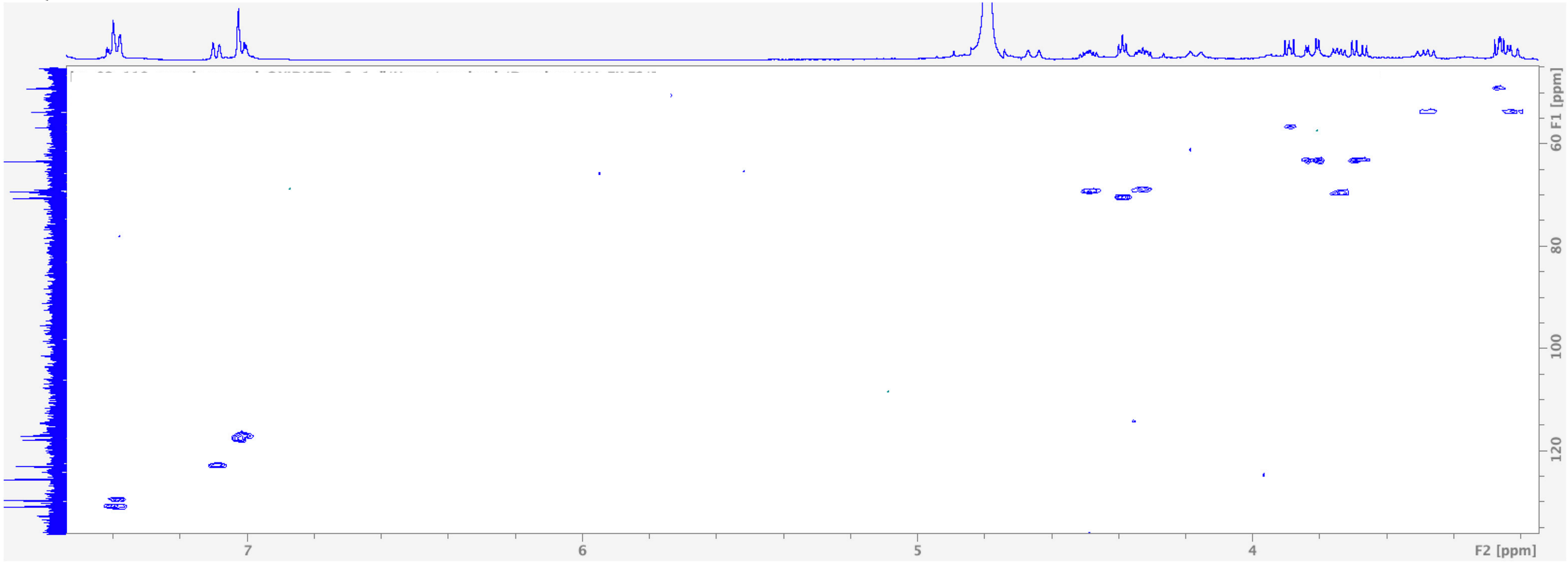

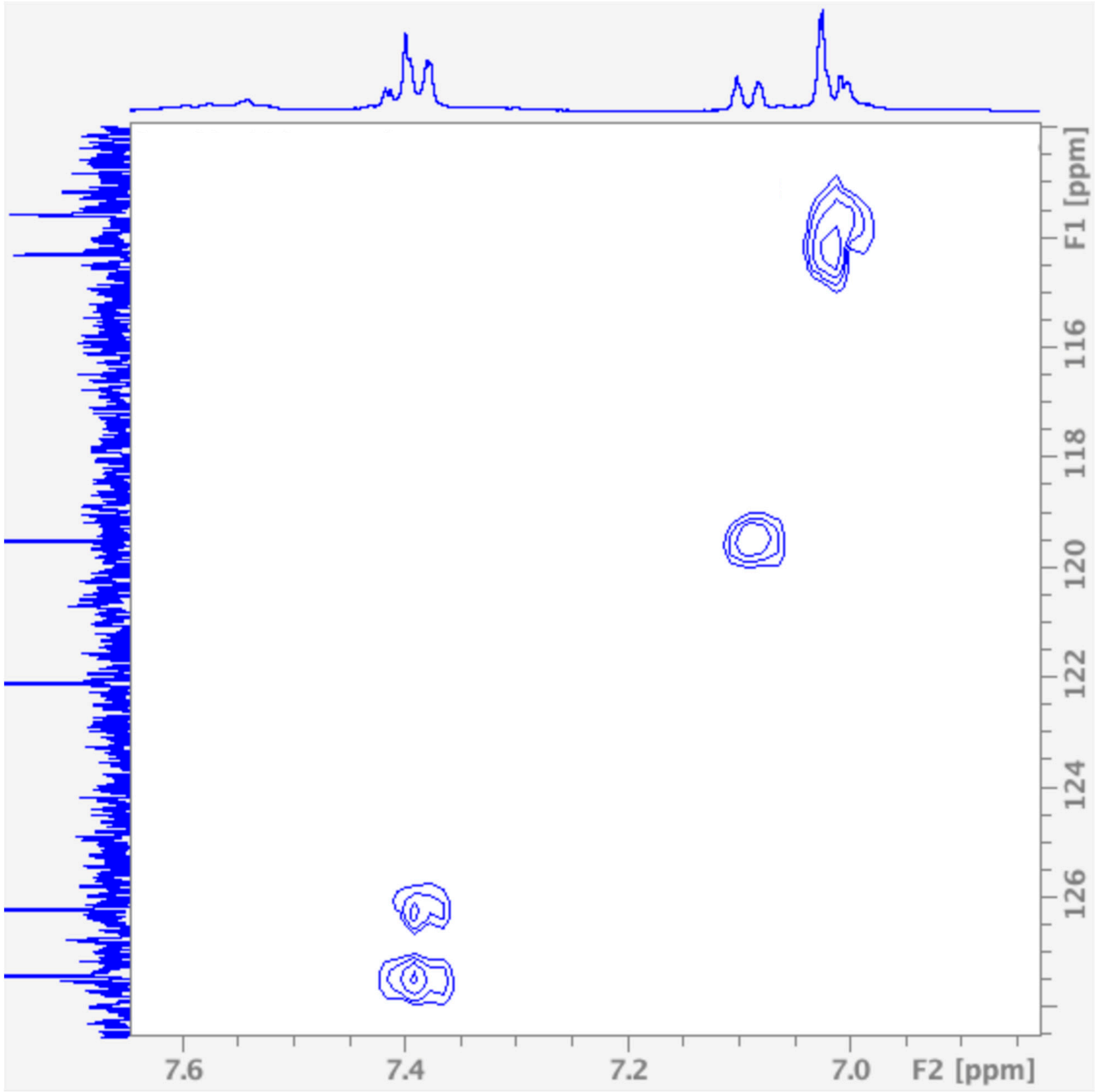

F1 [ppm]
116
118
120
122
124
126
7.6
7.4
7.2
7.0
F2 [ppm]

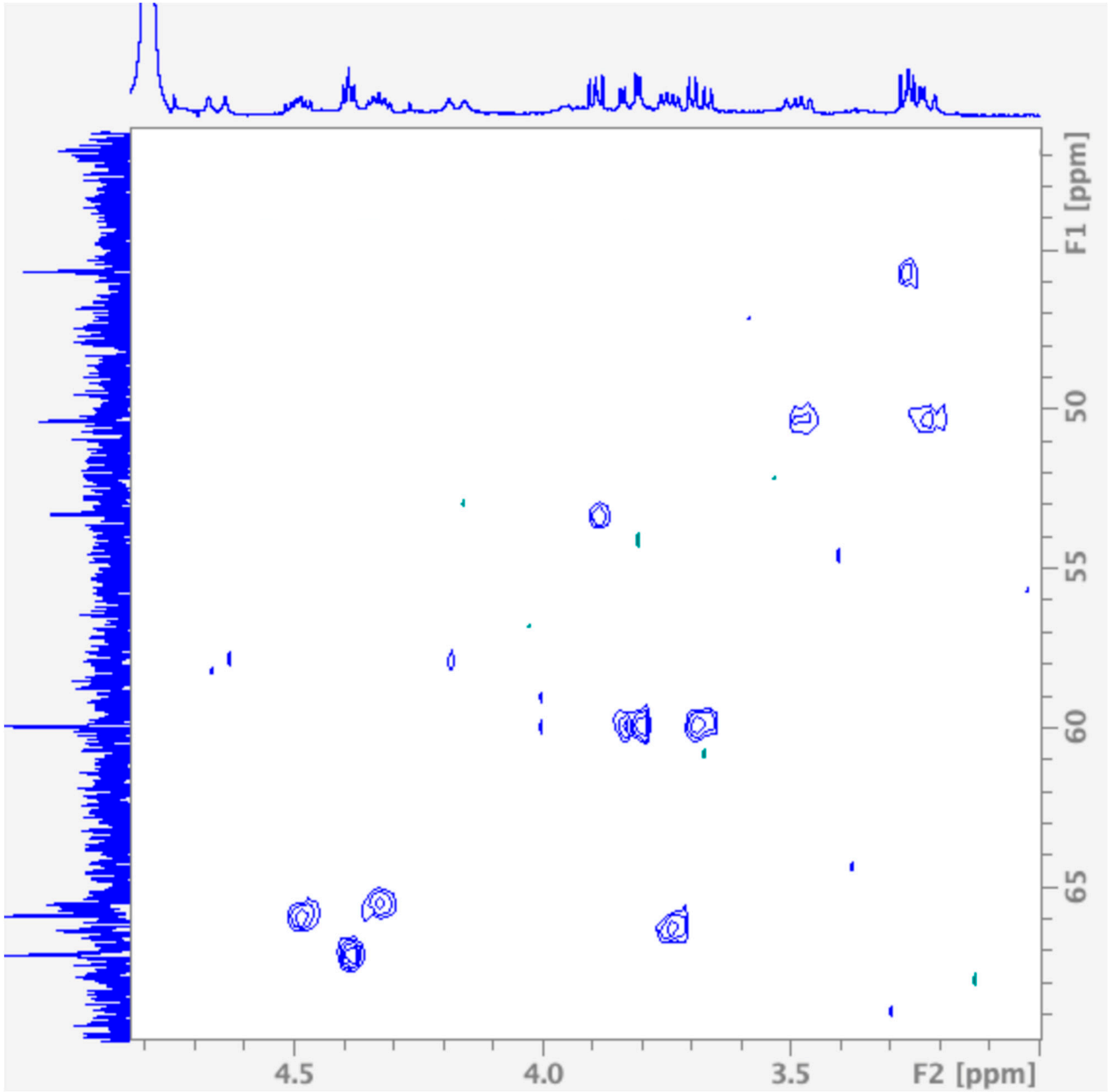

F1 [ppm]
50
55
60
65
4.5
4.0
3.5
F2 [ppm]

NMR Experimental details (*Acetic acid-d*[6])

	meta 6	meta 7
^{1}H	F2 - Acquisition Parameters<br>Date_ 20171012<br>Time 12.13<br>INSTRUM spect<br>PROBHD 5 mm PABBO BB-<br>PULPROG zg<br>TD 48076<br>SOLVENT Acetic<br>NS 8<br>DS 2<br>SWH 6002.401 Hz<br>FIDRES 0.124852 Hz<br>AQ 4.0047307 sec<br>RG 119.51<br>DW 83.300 usec<br>DE 16.70 usec<br>TE 298.1 K<br>D1 5.00000000 sec<br>TD0 1<br><br>======== CHANNEL f1 ========<br>SFO1 400.1320007 MHz<br>NUC1 1H<br>P1 15.00 usec<br>PLW1 12.50300026 W<br><br>F2 - Processing parameters<br>SI 32768<br>SF 400.1300097 MHz<br>WDW EM<br>SSB 0<br>LB 0.30 Hz<br>GB 0<br>PC 1.00	F2 - Acquisition Parameters<br>Date_ 20180427<br>Time 14.38<br>INSTRUM spect<br>PROBHD 5 mm PABBO BB-<br>PULPROG zg<br>TD 48076<br>SOLVENT D2O<br>NS 8<br>DS 2<br>SWH 6002.401 Hz<br>FIDRES 0.124852 Hz<br>AQ 4.0047307 sec<br>RG 107<br>DW 83.300 usec<br>DE 16.70 usec<br>TE 298.2 K<br>D1 5.00000000 sec<br>TD0 1<br><br>======== CHANNEL f1 ========<br>SFO1 400.1320007 MHz<br>NUC1 1H<br>P1 15.00 usec<br>PLW1 12.50300026 W<br><br>F2 - Processing parameters<br>SI 32768<br>SF 400.1299645 MHz<br>WDW EM<br>SSB 0<br>LB 0.30 Hz<br>GB 0<br>PC 1.00

^{13}C	F2 - Acquisition Parameters<br>Date_ 20171015<br>Time 1.56<br>INSTRUM spect<br>PROBHD 5 mm PABBO BB-<br>PULPROG zgpg30<br>TD 65536<br>SOLVENT Acetic<br>NS 4000<br>DS 4<br>SWH 22058.824 Hz<br>FIDRES 0.336591 Hz<br>AQ 1.4854827 sec<br>RG 192.58<br>DW 22.667 usec<br>DE 6.50 usec<br>TE 298.2 K<br>D1 2.00000000 sec<br>D11 0.03000000 sec<br>TD0 1<br><br>======== CHANNEL f1 ========<br>SFO1 100.6223253 MHz<br>NUC1 13C<br>P1 9.80 usec<br>PLW1 60.95399857 W<br><br>======== CHANNEL f2 ========<br>SFO2 400.1316005 MHz<br>NUC2 1H<br>CPDPRG[2 waltz16<br>PCPD2 90.00 usec<br>PLW2 12.50300026 W<br>PLW12 0.34731001 W<br>PLW13 0.28132001 W<br><br>F2 - Processing parameters<br>SI 65536<br>SF 100.6126556 MHz<br>WDW no<br>SSB 0<br>LB 0 Hz<br>GB 0<br>PC 1.40	F2 - Acquisition Parameters<br>Date_ 20180428<br>Time 16.41<br>INSTRUM spect<br>PROBHD 5 mm PABBO BB-<br>PULPROG zgpg30<br>TD 65536<br>SOLVENT D2O<br>NS 3000<br>DS 4<br>SWH 22058.824 Hz<br>FIDRES 0.336591 Hz<br>AQ 1.4854827 sec<br>RG 42.64<br>DW 22.667 usec<br>DE 6.50 usec<br>TE 298.1 K<br>D1 2.00000000 sec<br>D11 0.03000000 sec<br>TD0 1<br><br>======== CHANNEL f1 ========<br>SFO1 100.6223253 MHz<br>NUC1 13C<br>P1 9.80 usec<br>PLW1 60.95399857 W<br><br>======== CHANNEL f2 ========<br>SFO2 400.1316005 MHz<br>NUC2 1H<br>CPDPRG[2 waltz16<br>PCPD2 90.00 usec<br>PLW2 12.50300026 W<br>PLW12 0.34731001 W<br>PLW13 0.28132001 W<br><br>F2 - Processing parameters<br>SI 65536<br>SF 100.6130962 MHz<br>WDW EM<br>SSB 0<br>LB 1.00 Hz<br>GB 0<br>PC 1.40

^{11}B	F2 - Acquisition Parameters<br>Date_ 20171013<br>Time 6.56<br>INSTRUM spect<br>PROBHD 5 mm PABBO BB-<br>PULPROG zg<br>TD 65536<br>SOLVENT Acetic<br>NS 128<br>DS 4<br>SWH 25510.203 Hz<br>FIDRES 0.389255 Hz<br>AQ 1.2845056 sec<br>RG 192.58<br>DW 19.600 usec<br>DE 6.50 usec<br>TE 298.1 K<br>D1 1.00000000 sec<br>TD0 1<br><br>======== CHANNEL f1 ========<br>SFO1 128.3776052 MHz<br>NUC1 11B<br>P1 17.05 usec<br>PLW1 11.69499969 W<br><br>F2 - Processing parameters<br>SI 32768<br>SF 128.3776052 MHz<br>WDW EM<br>SSB 0<br>LB 1.00 Hz<br>GB 0<br>PC 1.40	F2 - Acquisition Parameters<br>Date_ 20180428<br>Time 17.05<br>INSTRUM spect<br>PROBHD 5 mm PABBO BB-<br>PULPROG zg<br>TD 65536<br>SOLVENT D2O<br>NS 128<br>DS 4<br>SWH 25510.203 Hz<br>FIDRES 0.389255 Hz<br>AQ 1.2845056 sec<br>RG 192.58<br>DW 19.600 usec<br>DE 6.50 usec<br>TE 298.1 K<br>D1 1.00000000 sec<br>TD0 1<br><br>======== CHANNEL f1 ========<br>SFO1 128.3776052 MHz<br>NUC1 11B<br>P1 17.05 usec<br>PLW1 11.69499969 W<br><br>F2 - Processing parameters<br>SI 32768<br>SF 128.3776052 MHz<br>WDW EM<br>SSB 0<br>LB 1.00 Hz<br>GB 0<br>PC 1.40

**DEPT**		F2 - Acquisition Parameters<br>Date_ 20180427<br>Time 14.56<br>INSTRUM spect<br>PROBHD 5 mm PABBO BB-<br>PULPROG deptqgpsp<br>TD 65536<br>SOLVENT D2O<br>NS 256<br>DS 4<br>SWH 22058.824 Hz<br>FIDRES 0.336591 Hz<br>AQ 1.4854827 sec<br>RG 192.58<br>DW 22.667 usec<br>DE 6.50 usec<br>TE 298.2 K<br>CNST2 145.0000000<br>CNST12 1.5000000<br>D1 2.00000000 sec<br>D2 0.00344828 sec<br>D12 0.00002000 sec<br>D16 0.00020000 sec<br>TD0 1<br><br>======== CHANNEL f1 ========<br>SFO1 100.6223258 MHz<br>NUC1 13C<br>P1 9.80 usec<br>P13 2000.00 usec<br>PLW0 0 W<br>PLW1 60.95399857 W<br>SPNAM[5] Crp60comp.4<br>SPOAL5 0.500<br>SPOFFS5 0 Hz<br>SPW5 8.94419956 W<br><br>======== CHANNEL f2 ========<br>SFO2 400.1316005 MHz<br>NUC2 1H<br>CPDPRG[2 waltz16<br>P0 22.50 usec<br>P3 15.00 usec<br>P4 30.00 usec<br>PCPD2 90.00 usec<br>PLW2 12.50300026 W<br>PLW12 0.34731001 W<br><br>====== GRADIENT CHANNEL =====<br>GPNAM[1] SMSQ10.32<br>GPNAM[2] SMSQ10.32<br>GPNAM[3] SMSQ10.32<br>GPZ1 31.00 %<br>GPZ2 31.00 %<br>GPZ3 31.00 %<br>P16 1000.00 usec<br><br>F2 - Processing parameters<br>SI 65536<br>SF 100.6127690 MHz<br>WDW EM<br>SSB 0<br>LB 1.00 Hz<br>GB 0<br>PC 1.40

**COSY**	F2 - Acquisition Parameters<br>Date_ 20171012<br>Time 12.39<br>INSTRUM spect<br>PROBHD 5 mm PABBO BB-<br>PULPROG cosygpqf<br>TD 2048<br>SOLVENT Acetic<br>NS 1<br>DS 8<br>SWH 4807.692 Hz<br>FIDRES 2.347506 Hz<br>AQ 0.2129920 sec<br>RG 192.58<br>DW 104.000 usec<br>DE 6.50 usec<br>TE 298.1 K<br>D0 0.00000300 sec<br>D1 1.48689198 sec<br>D13 0.00000400 sec<br>D16 0.00020000 sec<br>IN0 0.00020800 sec<br><br>======== CHANNEL f1 ========<br>SFO1 400.1322007 MHz<br>NUC1 1H<br>P0 15.00 usec<br>P1 15.00 usec<br>PLW1 12.50300026 W<br><br>====== GRADIENT CHANNEL =====<br>GPNAM[1] SMSQ10.100<br>GPZ1 10.00 %<br>P16 1000.00 usec<br><br>F1 - Acquisition parameters<br>TD 128<br>SFO1 400.1322 MHz<br>FIDRES 75.120193 Hz<br>SW 12.015 ppm<br>FnMODE QF<br><br>F2 - Processing parameters<br>SI 1024<br>SF 400.1300096 MHz<br>WDW SINE<br>SSB 0<br>LB 0 Hz<br>GB 0<br>PC 1.40<br><br>F1 - Processing parameters<br>SI 1024<br>MC2 QF<br>SF 400.1300090 MHz<br>WDW SINE<br>SSB 0<br>LB 0 Hz<br>GB 0	F2 - Acquisition Parameters<br>Date_ 20180427<br>Time 14.58<br>INSTRUM spect<br>PROBHD 5 mm PABBO BB-<br>PULPROG cosygpqf<br>TD 2048<br>SOLVENT D2O<br>NS 1<br>DS 8<br>SWH 4807.692 Hz<br>FIDRES 2.347506 Hz<br>AQ 0.2129920 sec<br>RG 192.58<br>DW 104.000 usec<br>DE 6.50 usec<br>TE 298.0 K<br>D0 0.00000300 sec<br>D1 1.48689198 sec<br>D13 0.00000400 sec<br>D16 0.00020000 sec<br>IN0 0.00020800 sec<br><br>======== CHANNEL f1 ========<br>SFO1 400.1322007 MHz<br>NUC1 1H<br>P0 15.00 usec<br>P1 15.00 usec<br>PLW1 12.50300026 W<br><br>====== GRADIENT CHANNEL =====<br>GPNAM[1] SMSQ10.100<br>GPZ1 10.00 %<br>P16 1000.00 usec<br><br>F1 - Acquisition parameters<br>TD 128<br>SFO1 400.1322 MHz<br>FIDRES 75.120193 Hz<br>SW 12.015 ppm<br>FnMODE QF<br><br>F2 - Processing parameters<br>SI 1024<br>SF 400.1299640 MHz<br>WDW SINE<br>SSB 0<br>LB 0 Hz<br>GB 0<br>PC 1.40<br><br>F1 - Processing parameters<br>SI 1024<br>MC2 QF<br>SF 400.1299640 MHz<br>WDW SINE<br>SSB 0<br>LB 0 Hz<br>GB 0

HSQC		
	F2 - Acquisition Parameters	F2 - Acquisition Parameters
	Date_ 20171012	Date_ 20180428
	Time 13.02	Time 16.44
	INSTRUM spect	INSTRUM spect
	PROBHD 5 mm PABBO BB-	PROBHD 5 mm PABBO BB-
	PULPROG hsqcetgpsisp2.2	PULPROG hsqcetgpsisp2.2
	TD 2048	TD 2048
	SOLVENT Acetic	SOLVENT D2O
	NS 2	NS 2
	DS 16	DS 16
	SWH 5341.880 Hz	SWH 5341.880 Hz
	FIDRES 2.608340 Hz	FIDRES 2.608340 Hz
	AQ 0.1916928 sec	AQ 0.1916928 sec
	RG 192.58	RG 192.58
	DW 93.600 usec	DW 93.600 usec
	DE 6.50 usec	DE 6.50 usec
	TE 298.3 K	TE 298.0 K
	CNST2 145.0000000	CNST2 145.0000000
	CNST17 -0.5000000	CNST17 -0.5000000
	D0 0.00000300 sec	D0 0.00000300 sec
	D1 1.50000000 sec	D1 1.50000000 sec
	D4 0.00172414 sec	D4 0.00172414 sec
	D11 0.03000000 sec	D11 0.03000000 sec
	D16 0.00020000 sec	D16 0.00020000 sec
	D24 0.00086207 sec	D24 0.00086207 sec
	IN0 0.00003000 sec	IN0 0.00003000 sec
	======== CHANNEL f1 ========	======== CHANNEL f1 ========
	SFO1 400.1324057 MHz	SFO1 400.1324057 MHz
	NUC1 1H	NUC1 1H
	P1 15.00 usec	P1 15.00 usec
	P2 30.00 usec	P2 30.00 usec
	P28 1000.00 usec	P28 1000.00 usec
	PLW1 12.50300026 W	PLW1 12.50300026 W
	======== CHANNEL f2 ========	======== CHANNEL f2 ========
	SFO2 100.6202713 MHz	SFO2 100.6202713 MHz
	NUC2 13C	NUC2 13C
	CPDPRG[2 bi_p5m4sp_4sp.2	CPDPRG[2 bi_p5m4sp_4sp.2
	P3 9.80 usec	P3 9.80 usec
	P14 500.00 usec	P14 500.00 usec
	P24 2000.00 usec	P24 2000.00 usec
	P63 1500.00 usec	P63 1500.00 usec
	PLW0 0 W	PLW0 0 W
	PLW2 60.95399857 W	PLW2 60.95399857 W
	PLW12 0.91469002 W	PLW12 0.91469002 W
	SPNAM[3] Crp60,0.5,20.1	SPNAM[3] Crp60,0.5,20.1
	SPOAL3 0.500	SPOAL3 0.500
	SPOFFS3 0 Hz	SPOFFS3 0 Hz
	SPW3 8.94419956 W	SPW3 8.94419956 W
	SPNAM[7] Crp60comp.4	SPNAM[7] Crp60comp.4
	SPOAL7 0.500	SPOAL7 0.500
	SPOFFS7 0 Hz	SPOFFS7 0 Hz
	SPW7 8.94419956 W	SPW7 8.94419956 W
	SPNAM[14 Crp32,1.5,20.2	SPNAM[14 Crp32,1.5,20.2
	SPOAL14 0.500	SPOAL14 0.500
	SPOFFS14 0 Hz	SPOFFS14 0 Hz
	SPW14 3.81620002 W	SPW14 3.81620002 W
	SPNAM[31 Crp32,1.5,20.2	SPNAM[31 Crp32,1.5,20.2
	SPOAL31 0.500	SPOAL31 0.500
	SPOFFS31 0 Hz	SPOFFS31 0 Hz
	SPW31 0.95405000 W	SPW31 0.95405000 W
	====== GRADIENT CHANNEL =====	====== GRADIENT CHANNEL =====
	GPNAM[1] SMSQ10.100	GPNAM[1] SMSQ10.100
	GPNAM[2] SMSQ10.100	GPNAM[2] SMSQ10.100
	GPNAM[3] SMSQ10.100	GPNAM[3] SMSQ10.100
	GPNAM[4] SMSQ10.100	GPNAM[4] SMSQ10.100
	GPZ1 80.00 %	GPZ1 80.00 %
	GPZ2 20.10 %	GPZ2 20.10 %
	GPZ3 11.00 %	GPZ3 11.00 %
	GPZ4 -5.00 %	GPZ4 -5.00 %
	P16 1000.00 usec	P16 1000.00 usec
	P19 600.00 usec	P19 600.00 usec
	F1 - Acquisition parameters	F1 - Acquisition parameters
	TD 256	TD 256
	SFO1 100.6203 MHz	SFO1 100.6203 MHz
	FIDRES 130.208328 Hz	FIDRES 130.208328 Hz
	SW 165.639 ppm	SW 165.639 ppm
	FnMODE Echo-Antiecho	FnMODE Echo-Antiecho
	F2 - Processing parameters	F2 - Processing parameters
	SI 1024	SI 1024
	SF 400.1300087 MHz	SF 400.1299646 MHz
	WDW QSINE	WDW QSINE
	SSB 2	SSB 2
	LB 0 Hz	LB 0 Hz
	GB 0	GB 0
	PC 1.40	PC 1.40
	F1 - Processing parameters	F1 - Processing parameters
	SI 1024	SI 1024
	MC2 echo-antiecho	MC2 echo-antiecho
	SF 100.6126505 MHz	SF 100.6130928 MHz
	WDW QSINE	WDW QSINE
	SSB 2	SSB 2
	LB 0 Hz	LB 0 Hz
	GB 0	GB 0
